# “*Maybe we should think outside the box*?*”* prioritisation of issues with UK not-for-profit canine health and welfare research funding using Delphi expert consensus and gap analysis

**DOI:** 10.1371/journal.pone.0313735

**Published:** 2024-12-04

**Authors:** Alison M. Skipper, Rowena M. A. Packer, Dan G. O’Neill

**Affiliations:** 1 Department of Pathobiology and Population Sciences, The Royal Veterinary College, Hawkshead Lane, North Mymms, Hatfield, Hertfordshire, United Kingdom; 2 Department of Clinical Science and Services, The Royal Veterinary College, Hawkshead Lane, North Mymms, Hatfield, Hertfordshire, United Kingdom; University of Wisconsin-La Crosse, UNITED STATES OF AMERICA

## Abstract

Over fifty participants, who together possessed broad research, veterinary and front-line expertise from across the canine health and welfare sector, contributed to a modified Delphi study to identify the highest priority research topics in UK canine health and welfare, the highest priorities for future research approaches, and the highest priorities for future reform in research processes and infrastructure, through group consensus. Further analysis also compared the prioritisation of selected research topics to the actual levels of research funding they previously received, through comparison with historical data. Most of the identified highest priority issues relating to canine health and welfare and its research concerned various aspects of the human-canine relationship, such as ownership or behavioural issues. Participants strongly emphasised the complexity of interrelated factors that impact the welfare of both dogs and people. Research topics identified as previously ‘most underfunded’ all concerned real-world canine welfare issues, particularly emphasising the breeding and supply of dogs. A supplementary analysis of historical research funding (2012–2022) for common chronic disorders in primary care practice, another identified highest priority topic, identified periodontal disease, anal sac disorders, overgrown nails and patellar luxation as the ‘most underfunded’ conditions. Most of the identified highest priority research approaches and methodologies concerned real-world design and execution aspects of canine health and welfare research, such as impact and engagement, with a strong focus on research investigating the human factors in canine welfare. Aspects of research funding infrastructure that were considered highest priority for future change mostly concerned increased transparency of funding processes and increased collaboration between stakeholder groups throughout the funding sector, which was strongly supported. Overall, these findings emphasise the importance of considering and including human factors and real-world impact, where appropriate, as key elements for optimising the relevance of canine health and welfare research.

## 1. Introduction

Dogs are popular as companion animals, with an estimated UK population of 11 million in 2023, and many research projects investigating their health and welfare [1]. On Google Scholar, a search using the term ‘dog health’ returns over 75,000 articles published globally in 2022 alone. However, despite this extensive and wide-ranging research activity, there has been little previous formal investigation of what research areas should be prioritised to maximise health and welfare improvement of this species in the modern world [2–4]. Given that research funding, infrastructure and staff are all finite resources, there is clear value in developing a strategy for the future allocation of research funding to maximise impact and benefit for the dog. Such a strategy would require detailed background knowledge of the current sources and value of research funding within this sector and of its historical distribution. It would also require informed understanding of what areas are considered high (or highest) priority for future investigation and of what research approaches are considered most useful. Finally, it would require a commitment from funders to direct future funding towards the identified high-priority areas for research, particularly those that are currently most underfunded, and, if possible, to address any logistical problems with current research funding processes. Effective implementation of this strategy would also require collaboration between funding organisations and communication with other sector stakeholders, to ensure that multiple perspectives are included in these assessments and contribute to these decisions, where appropriate.

The current paper is part of a larger research project on UK canine research funding intended to explore these sector problems and fill these gaps, which was jointly commissioned by four UK not-for-profit animal-directed funders of canine health and welfare research. The first study in this larger research project investigated UK not-for-profit funding of canine health and welfare from 2012–2022, identifying £57.6 million of total funding [5]. It mapped the funding landscape to calculate the market share contributed by different wide-scope (e.g. UK government research councils) and animal-directed (e.g. charitable) not-for profit funding bodies, and described the distribution of this funding across different destination institutions and research fields. Customised metrics were also developed and used to evaluate ‘benefit for the dog’ and ‘pathway to impact’ for research projects across the resulting dataset.

This prior work built a foundation for subsequent research by producing a detailed data analysis of the sources, amount and distribution of UK not-for-profit funding of canine health and welfare research. However, the initial study did not investigate what research was most needed, so did not consider whether and how future funding could be deployed to maximise benefits to canine health and welfare. This latter objective was addressed by the second phase of the overall research project, which is described in this paper. This second phase study involved two linked stages of investigation, which both contributed to identifying highest priority current gaps in knowledge, funding and processes within this sector. The first stage used a modified Delphi approach to establish stakeholder consensus on the current highest priority issues within canine health and welfare and its research funding in the UK. These Delphi findings were then analysed against the funding dataset from the first phase of the project to identify which highest priority research topics previously received least (and most) funding. The Delphi study also revealed the highest priority research approaches and methodologies and the highest priority suggested changes to current research processes and infrastructure, as determined by participant consensus, thus providing broad evidence to inform future reforms of funding processes and aims.

The Delphi study technique is ‘a method for … allowing a group of individuals, as a whole, to deal with a complex problem’ [6]. This approach is widely used in health sciences and is particularly suitable for establishing a collective opinion on topics that are inherently subjective, such as social phenomena or matters of policy, especially when views may vary [7]. Classically, participants with a spread of relevant expertise are asked to contribute their opinions on the study topic and thereafter revise their views following exposure to the opinions of the wider group, so that an overall consensus view is progressively reached [8]. Although the original Delphi studies used several anonymised rounds of structured communication, there are now many methodological variations and aims. For example, studies may seek to map diversity of opinion or to brainstorm solutions to a problem, rather than to reach a single expert consensus [7]. Moreover, Delphi studies have varied greatly in the number and selection of their participants, how many discussion rounds are undertaken and how consensus is determined, with an inevitable trade-off between the more robust output from a study with more respondents and more iterative rounds of discussion and the higher workload, greater time commitment and higher drop-out rates that a larger, longer investigation often involves [9,10]. Recently, many studies have adopted a ‘modified Delphi’ approach, a term which usually refers to a heterogenous approach that combines elements of ‘classic’ anonymous consultation (typically through questionnaires) with at least one phase of face-to-face group discussion [11]. This structure is thought to produce superior outputs by harnessing the power and insight offered by group problem-solving [12].

The term ‘gap analysis’ is a broad term used to describe the investigation of many types of potential gaps between expected, preferred or idealised situations and the actual situation—for example, a lack of congruence between organisational and societal expectations of a business issue such as environmental sustainability [13]. Gap analysis has been defined as ‘a process to identify … what differences exist between [the] … current situation and “what ought to be” in place’ [14]. For example, healthcare organisations might conduct an audit to identify gaps between best practice evidence-based protocols for patient interventions and data recording and those that are actually used across their treatment centres [15]. In academia, gap analysis to identify subject areas in need of more research frequently relies on a literature review, which may be methodologically problematic and is inherently retrospective, with a time lag caused by the publication process [16]. One alternative approach for such a research gap analysis involves a Delphi-type study where a group of experts iteratively develop a consensus on what research questions most urgently need answering within their field [17]. This Delphi-type approach is considered particularly suitable to identify research gaps within clinical healthcare, because it has the advantage that participants are collating their personal subject knowledge, which is already both highly specific and up-to-date; it also enables participants to build their own understandings through shared information and collaborative thinking [18].

Animal welfare is a complex topic where scientific evidence is inevitably brought into dialogue with subjective opinion and the current zeitgeist [19]. Consequently, the Delphi technique is well-established as a useful methodology to investigate issues around animal welfare [20]. A growing body of work has used the Delphi approach to prioritise broader aspects of canine welfare in the UK [3,21,22]. However, those studies did not consider canine health in detail and no previous studies have explored research funding or processes, so that there has been no prior formal attempt to construct an evidence-based gap analysis for future funding deployment or infrastructure reform in the canine health and welfare sector.

The current modified Delphi study aimed to address this deficit by obtaining stakeholder consensus on the highest priorities for future funding deployment and infrastructure reform in canine health and welfare research. This comprised the first stage of gap analysis, which identified multiple points of concern across this sector that were deemed in need of greater attention or process revision, and organised these data into inductively derived problem categories suitable for further scrutiny. Secondly, the current study also aimed to offer the first critical gap analysis of funding distribution in the canine health and welfare sector, by comparing the highest priority Delphi prioritisation scores with the relevant funding awarded in the historical dataset previously described [5]. The study thus provides the first evidence-based sector investigation that can inform the future prioritisation of UK not-for-profit canine health and welfare research funding and its infrastructure.

## 2. Method

### 2.1 Ethical approval

This study was granted ethics approval by the Social Science Research Ethical Review Board at the Royal Veterinary College (URN SR2023–0106).

### 2.2 Overall methodology

The current study used a modified group Delphi technique to obtain consensus priority scores on a wide range of topics related to canine health and welfare and its not-for-profit funding in the UK. It involved 59 participants, deliberately selected to offer a broad range of sector-relevant expertise. It included two rounds of investigation: an anonymised online questionnaire where participants suggested the points of concern that they considered most important to various aspects of canine health and welfare and its research processes and funding, followed by an in-person workshop which asked the same participants, within arranged discussion groups, to collaboratively prioritise (and, where necessary, modify) an extensive list of these previously suggested points of concern. The discussion transcripts and raw scores were then collated and organised to synthesise a consensus overview of the highest priority points of concern in UK canine health and welfare research and its funding. The consensus highest priority points of concern in canine health and welfare were then compared with historical not-for-profit funding from the previously described historical dataset [5], to establish which points of concern had previously been most underfunded relative to their prioritisation scores and should therefore be considered highest priority for increased future funding.

**Fig 1:**
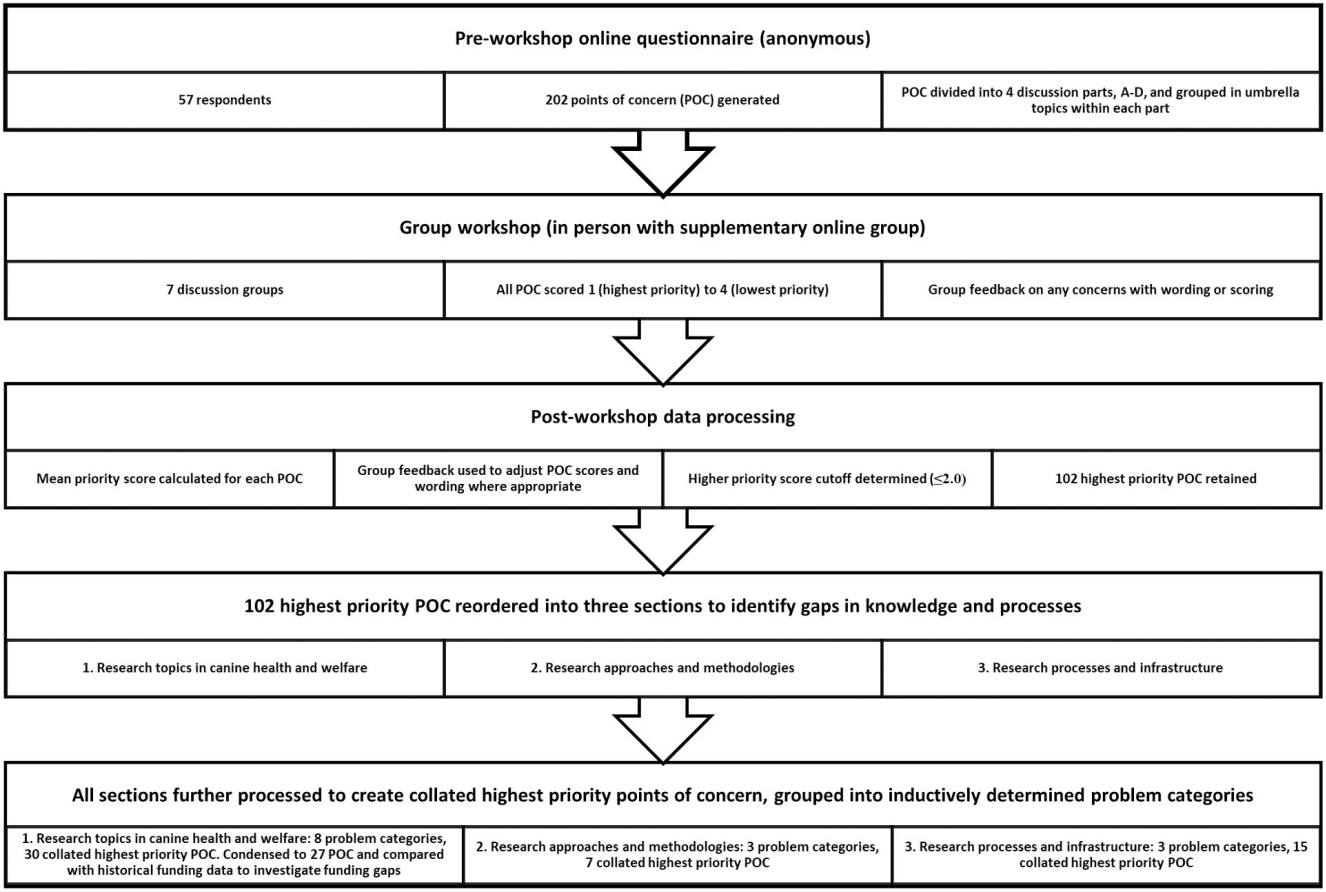
Flowchart of research processes used to explore canine health and welfare research and its UK not-for-profit funding, using modified Delphi expert consensus and subsequent comparison with historical funding data to provide a gap analysis for future prioritisation of issues in this sector.

The flowchart in Fig 1 summarises the stages of data collection and processing that comprised the overall methodology for this modified Delphi study. These processes are also described briefly below and in more detail in S1 Appendix.

### 2.3 Data collection

#### 2.3.1. Recruitment of study participants

Study participants were based in the UK and were chosen to collectively offer broad expertise in canine health and welfare, to ensure that a wide range of relevant viewpoints were included, thus maximising the capture of varied informed opinions and minimising bias. They included veterinary professionals involved in first opinion and referral practice and research; researchers engaged in biological and social sciences and humanities; representatives of funding organisations; people who worked for dog-relevant charitable organisations; and dog breeders. The recruitment process deliberately aimed to recruit between 50 and 60 participants in total. The number of participants was chosen to ensure that each skillset would be represented by multiple people (many individual people also had multiple skillsets). Recruitment began through personal networking by the authorship team and was then expanded through a ‘snowball’ system of asking direct contacts to propose other people with relevant expertise from within their own networks. A tally was kept of recruited participants with different types of expertise; if one invited person was unable to participate, another person with similar expertise was approached in their stead. From 12 April 2023 onwards, possible participants were informed of the date of the workshop so they could plan their diaries, but formal recruitment was only confirmed when data collection began on 26 July 2023.

#### 2.3.2. Pre-workshop online questionnaire

On 26 July 2023, six weeks before the workshop, data collection began by circulating an online pre-workshop questionnaire to study participants via SurveyMonkey. The full questionnaire is provided in S1 Appendix. This anonymised questionnaire asked participants to provide their background demographic information and to describe their sector expertise and experience. It then asked six broad questions about the canine health and welfare sector and its research, which were each answerable via an unlimited free-text field. It concluded with a consent form. Following several reminders, the questionnaire was closed on 30 August 2023, one week before the workshop.

#### 2.3.3. Collation of responses from pre-workshop questionnaire

The demographic information provided by respondents was tabulated in Microsoft Excel. Using Microsoft Word, the free-text responses to the six questions were extracted, deconstructed into individual components and collated where necessary to create a long list of elicited points of concern. Using Microsoft Excel, these extracted points of concern were grouped into umbrella topics and divided into four parts to structure the discussion at the workshop. These four parts were:

A. What problems have the greatest negative impact on overall canine health and welfare?
B. Which are the most detrimental structural problems that exist in the current UK landscape of canine health and welfare funding?
C. What aspects of currently funded canine health and welfare research is it most important to change the amount of money directed to? (underfunded and overfunded were considered separately).
D. What are the most important changes needed in research funding to increase the positive impact for canine welfare?

**Fig 2:**
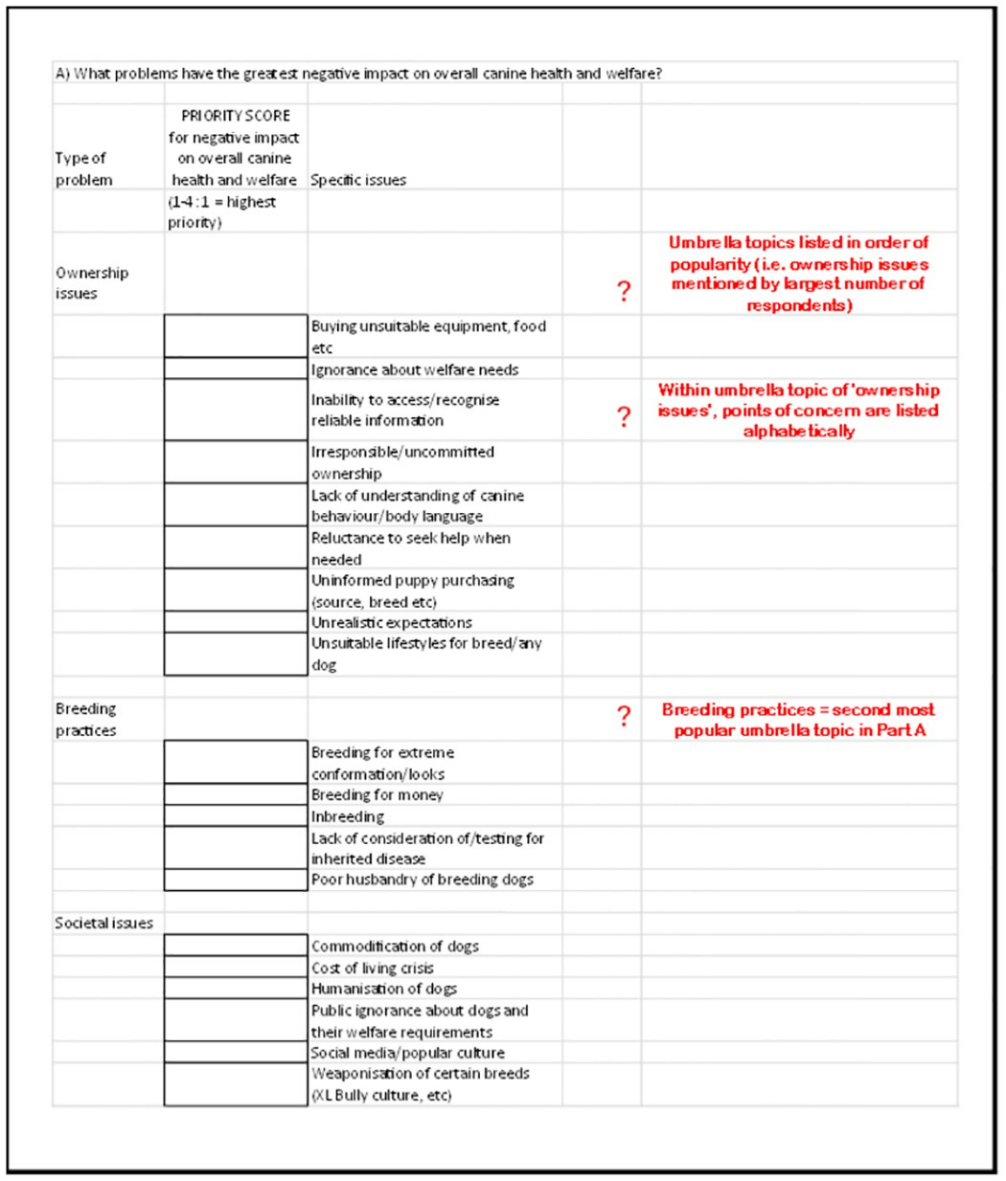
Excerpt from Excel discussion framework for the modified Delphi workshop discussion Part A, annotated in red to explain its structure.

Within each part A-D of the discussion framework, umbrella topics were sorted and listed from high to low according to the frequency with which respondents had mentioned them, and individual points of concern were listed alphabetically within each discussion topic. This process is described in more detail in S1 Appendix, and the full discussion framework is provided in sheets A-D in the S1 Dataset. This discussion framework was pre-circulated to all attendees a few days before the workshop to allow familiarisation with its content. An illustrative excerpt is provided in Fig 2. below.

#### 2.3.4. Group workshop

Immediately before the in-person workshop on 6 September 2023, confirmed attendees were sorted non-randomly into six discussion groups, deliberately selected to ensure as even a spread of expertise as possible between groups. For example, a single group of eight people might include a funder, a clinical researcher, a veterinarian in first opinion practice, a geneticist, a charity worker, a veterinary nurse and a breed community health volunteer, with individual people potentially representing multiple roles and skillsets. Attendees from the same organisation were split across groups where possible. Each group was allocated a session leader, who was either one of the authors of this paper or one of their postgraduate students or staff colleagues. All attendees were reminded that Chatham House rules applied [23]. Attendees were briefed on the purpose and scope of the research and asked to provide written consent for participation in and recording of the discussions. They were then asked to consider the various discussion framework parts within their allocated groups and to collaboratively score each point of concern from 1–4, where 1 was highest priority and 4 was lowest priority. This process is described in more detail in S1 Appendix. A supplementary online discussion session was held on 13 October 2023 to capture input for eight delegates who were unable to attend the in-person workshop on the day, making a total of seven discussion groups that contributed to the final analysis. All groups discussed parts A and D, but parts B and C were covered only by some groups, as explained in S1 Appendix.

### 2.4. Data processing

#### 2.4.1. Post-workshop data collation

All seven session recordings were manually transcribed by the lead researcher (AMS). Comments addressing each point of concern were extracted, compared across discussion groups, and summarised to a few sentences that captured the overall consensus viewpoint. Each point of concern summary and all individual discussion group priority scores were tabulated in Excel (Microsoft). A mean priority score was calculated for each point of concern from the scores of all participating discussion groups. This summarised and anonymised information is included in the S1 Dataset (see also S1 Appendix).

#### 2.4.2. Selection of highest priority points of concern

This overall process is discussed in detail in S1 Appendix. The mean priority score data were scrutinised to determine a suitable cut-off point for highest priority points of concern. A cut-off score of 2.0 was chosen across all discussion groups to differentiate between higher (≤ 2.0) and lower (> 2.0) priority points of concern. All points of concern with a mean priority score of > 2 (lower priority) were excluded from further analysis. This created an overall ‘highest priority’ list of 102 points of concern, 50.5% of the original total number. Some adjustments were made to the wording and/or score of a few highest priority points of concern during this process, in response to participant feedback.

#### 2.4.3. Reordering of highest priority points of concern into three themed sections

The 102 revised highest priority (mean priority score ≤ 2.0) points of concern were inductively regrouped into three sections according to the broad focus of each. These were:

1. Research topics in canine health and welfare, which included both dog-facing problems, such as emerging infectious diseases or long-term shelter welfare, and human factors, such as breeding for extreme conformation/looks or the cost-of-living crisis.
2. Research approaches and methodologies, such as ‘prospective studies’ or ‘social science research’.
3. Research processes and infrastructure, such as ‘support for early career researchers’ or ‘visibility of current funding patterns’.

### 2.5. Further processing of the three themed sections to produce gap analyses of these highest priority points of concern

Each of these three reordered sections was then further processed separately, as described briefly below and more fully in S1 Appendix.

#### 2.5.1. Research topics in canine health and welfare

The highest priority points of concern relating to research topics in canine health and welfare were scrutinised for overlap, collated if necessary, and divided into inductively determined meaningful problem categories, informed by the discussion transcripts and by logical categorisation of overlapping or related points of concern. Where collation was used, the resulting collated points of concern were awarded a collated mean priority score. Some highest priority Delphi points of concern were merged so that they could be mapped effectively to the historical research funding allocation previously described in the first phase of this research project [5]. Past funding that was relevant to the highest priority research topics as determined by the Delphi study analysis was then identified within the historical dataset, extracted, summed, and mapped onto each collated highest priority point of concern. Each research grant was allotted only once within each problem category but could appear in other problem categories if it was deemed relevant to more than one problem category. A priority score ranking and a total past funding ranking were calculated for each collated point of concern. The overall historical funding for each problem category was calculated and split by type of funder, separating wide-scope funders (UK Research and Innovation (UKRI) councils and the Wellcome Trust) from animal-directed funders. SPSS Statistics version 29.0 (IBM) was used to plot ranked associations between (collated) Delphi mean priority scores and past funding expenditure £, and thereby to perform a gap analysis that identified which highest priority points of concern had previously received comparatively less past funding, relative to their priority score, than others.

A different approach was used for a subsidiary gap analysis that further investigated one highest priority point of concern from the Delphi study, ‘common chronic conditions in primary care practice’. This used previously published research that reported the prevalence of different common conditions in UK primary care canine practice as an external data source to inform the analysis [24]. The conditions identified in this previous paper were scrutinised to identify and extract common chronic conditions. Where necessary, these conditions were condensed into grouped categories that mapped onto identified categories in the historical funding dataset, and the individual prevalences of these conditions was added to obtain a grouped category value. Using this method, ten common chronic conditions with a prevalence ≥ 1% were identified and compared with the historical dataset to identify the ‘actual’ total funding previously directed to each condition. A notional ‘fair’ total funding was also calculated for each condition, assuming that the overall research spend had been distributed equitably in proportion to the prevalence. By comparing the percentages of the overall research funding that was actually allocated against what would have been ‘fairly’ allocated, it was possible to identify which common chronic conditions were relatively underfunded or overfunded in the historical dataset.

#### 2.5.2. Research approaches and methodologies

Highest priority points of concern that concerned research approaches or methodologies were grouped together, collated, condensed into new inductively determined problem categories, and scrutinised for overarching analytical themes.

#### 2.5.3. Research processes and infrastructure

Highest priority points of concern that concerned structural or logistical aspects of research funding processes were also grouped together, collated, condensed into new inductively determined problem categories, and scrutinised for overarching analytical themes.

## 3. Results

Background demographic and data collection results are reported concisely below and in more detail in S1 Appendix.

### 3.1 Recruitment of study participants

Sixty people were selected and invited to complete the online anonymous pre-workshop questionnaire.

### 3.2 Pre-workshop online questionnaire

The pre-workshop questionnaire was completed by 57 people, representing a 95% response rate among the 60 people invited. The demographic characteristics of these people are provided in S1 Appendix. Their current and previous involvement in the canine health and welfare sector is shown in the ‘participant skillset’ sheet in S1 Dataset and described in S1 Appendix. The respondents’ answers to the free text survey questions were processed as previously described to produce 202 individual points of concern, grouped into umbrella discussion topics and divided into four discussion parts A-D (Fig 1).

### 3.3 Group workshop

In total, 59 people attended the seven discussion groups in the second round of the modified Delphi analysis. Except for a few points of concern that certain discussion groups deliberately declined to score, all discussion groups fully scored all points of concern in discussion parts A and D. Due to time constraints, most groups did not complete both B and C (see full details in S1 Dataset). The number of points of concern within each discussion part A-D scored by each discussion group are provided in S1 Appendix, together with an account of how different groups negotiated the time restriction.

### 3.4 Post-workshop data processing

For each point of concern, a mean priority score was calculated from the available individual priority scores, as shown on sheets A-D in S1 Dataset. The wording and/or prioritisation score was adjusted for nine points of concern, where indicated by group discussion consensus; this is discussed in S1 Appendix and shown in the S1 Dataset. A cut-off score of 2.0 was used to differentiate between higher and lower priority points of concern, as discussed earlier. This process created a ‘highest priority’ list of 102 points of concern, 50.5% of the original total of 202 (see ‘Highest priorities A-D, all’ in S1 Dataset). The 100 discarded points of concern, each with a mean priority score > 2, are shown on the spreadsheet ‘lower priority, A-D’ in the S1 Dataset.

### 3.5 Reordering of highest priority points of concern into three themed sections

The 102 highest priority points of concern were reordered into three sections, as described above: research topics in canine health and welfare (46 points of concern); research approaches and methodologies (23 points of concern); and research processes and infrastructure (33 points of concern). The full list (before further processing) is shown in the spreadsheet ‘highest priorities—grouped’ in S1 Dataset.

### 3.6 Further processing of the three themed sections to produce gap analyses of these highest priority points of concern

#### 3.6.1 Research topics in canine health and welfare

The points of concern within this section were scrutinised, collated where appropriate, and rearranged into inductively determined meaningful problem categories, informed by the discussion transcripts and by logical categorisation and/or collation of similar or overlapping issues. This process produced 30 collated points of concern, reorganised into eight problem categories (Table 1).

**Table 1:** Highest priority (mean priority score ≤ 2.0) research topics in canine health and welfare, as determined by the modified Delphi study, collated and organised by inductively derived problem categories.

Problem category	Type of problem	Original POC part (and score if subsequently collated)	Specific issues	Comments from participants, summarised	Overall (collated) mean priority score (scale 1–4; lower number = higher priority)
1) Canine behaviour	Canine behavioural issues	Two points from A: anxiety (1.43), socialisation (1.57); and one point from C: aggression/anxiety (2.00).	General behavioural issues	General consensus that issues such as anxiety, aggression, socialisation are all interconnected and important, so grouped together here.	1.67
	Human factors relating to canine behaviour	A	Human lifestyle impact on canine behaviour	All agreed highest priority, little discussion needed	1.00
		A	Lack of understanding of canine behaviour/body language	Most agreed highest priority, little discussion	1.29
		One point from A: impact of training methods (2.00); one from C: regulation of trainers/behaviourists (1.83).	Training methods and regulation of trainers/behaviourists	Agreed that lack of regulation of trainers/behaviourists and impact of aversive methods are high priority welfare issues.	1.92
2) Ownership issues	Owner ignorance	A	Inability to access/recognise reliable information	General agreement that this is a big problem. There is plenty of reliable information available, but it may be harder to find or understand than unreliable information.	1.43
		A	Ignorance about welfare needs	Some people, even committed owners, are genuinely unaware of welfare needs, while others know but don’t implement them adequately.	1.57
	Unsuitable or unrealistic ownership	A	Unrealistic expectations	Many different types of unrealistic expectations—a key contributing problem.	1.29
		A	Unsuitable lifestyles for breed/any dog	Generally regarded as major problem—not enough time for a dog, plus mismatches between activity levels/strength of owners and dogs.	1.29
		A	Uninformed/uncommitted ownership	Strong agreement that this is a huge problem, albeit not often deliberately irresponsible.	1.71
3) Societal issues	Societal	A	Public ignorance about dogs and their welfare requirements	Most groups scored this as high or very high priority with little discussion.	1.43
		One point from A: social media/popular culture (1.57); one from C: role of social media (1.50).	Social media/popular culture	Many groups considered social media a key ’root cause’ of multiple welfare issues.	1.54
		A	Cost of living crisis	Multiple charity workers said that the cost-of-living crisis has had a massive welfare impact.	1.57
		C	Dog bite attacks	Rated highest priority for more research (BEFORE 2023 announcement of new XL Bully measures legislation).	1.00
4) Breeding and supply issues	HBC (human behavioural change)	One point from A: uninformed puppy purchasing (1.00); one point from C: puppy buying behaviours (1.25).	Uninformed puppy buying behaviours	Generally rated a very high priority with little discussion—buyer ignorance plus too few well-bred puppies is a massive welfare issue.	1.13
	HBC	One point from A: breeding for extreme conformation/looks (1.00); one from C: conformation-related disease (1.50).	Breeding for extreme conformation/looks	Extreme conformation universally considered highest priority welfare issue; HBC to tackle demand for this also considered highest priority by some but less familiar to others.	1.25
	Infrastructure issues	One point from A: lack of regulatory enforcement for online trading (1.36); one from C: online puppy sales (1.33).	Online puppy trading and lack of regulatory enforcement	Major problem that facilitates impulse buying of puppies with poor traceability: regulatory enforcement also flagged as an issue.	1.35
	Societal	One point from A: exploitative breeding for profit (1.86); two from C: criminology of puppy trade (1.67), fertility clinics (2.00).	Exploitative breeding for profit	Also includes criminology of puppy trade, issues linked to assisted breeding/fertility clinics: ‘*the dark end of dog breeding’*.	1.84
	HBC	C	Exploring better dog breeding	HBC—how to increase the supply of healthy well-bred dogs—unanimously highest priority.	1.00
	Legal issues	A		Lack of EFFECTIVE breeding regulation (or its enforcement) considered a high priority issue.	1.57
	Non-clinical	C		Quantitative genetics—all groups rated this high priority. Broad relevance so high value, can offer huge benefits at population level and inform HBC, etc.	2.00
	Societal	C		Which breeders produce healthier dogs? Feeds into how to ensure bigger proportion of dogs are bred ethically.	2.00
5) Breed-related diseases (overall)	Canine physical disease—breed-related	A	Breed-related diseases	Overall, a high priority problem that affects a lot of dogs, even including non-pedigree or unregistered dogs.	1.36
6) Issues related to importation	Social issues	One point from A: issues related to importation (1.29); one from C: import trade (1.00).	Issues of welfare for dogs imported via problematic routes: street dogs, transport welfare, smuggling, dogs being bred for ’rescue’, etc.	All rated this highest/high priority because of serious welfare concerns for large numbers of dogs.	1.15
	Exotic infectious diseases	Two points from C: emerging infectious diseases (1.25) exotic disease modelling (1.75).	Emerging infectious diseases.	Rated this high priority because of increasing risk of zoonotic diseases; disease modelling given high priority.	1.50
7) Clinical practice	Social issues	A	Access/affordability of veterinary care	Mostly rated highest priority. Impact of staffing crisis?	1.14
	Ethics	Two points from C: interventions that extend quantity but not quality of life (1.00), veterinary overtreatment/euthanasia decisions (1.50).	Veterinary overtreatment/euthanasia decisions	Various high priority issues collated: defensive medicine; high-profile cutting edge surgical interventions; long-term medical treatment of animals to extend quantity but not quality of life; reluctance to euthanise.	1.25
	Clinical welfare	C	Welfare impact of specific real-world problems	Workshop example was paralysed dogs in carts, but no intention to limit it to this specific problem.	1.33
	Canine physical disease -general	One point from A: very common diseases (1.14); one point from B: primary care veterinary work (2.00); two points from C: general (primary care) (1.00), common conditions, especially chronic (1.25).	Common diseases in primary care practice, especially chronic disorders	Generally agreed these problems have a huge welfare impact as they affect many animals for a long time, and that previous lack of primary care research has overlooked an area where funding can have a major impact.	1.35
	Clinical	C	Long-term health impacts of medication/diet	More emphasis given to impact of nutrition and diet than of medication.	2.00
8) Shelter welfare	Shelter medicine	C	Long term shelter welfare	Major issue but for few dogs	2.00

The 30 collated points of concern listed in Table 1 were compared with the dataset of historical funding patterns created during the first phase of this research, as shown in Table 2. The funding column in Table 2 displays the total relevant funding identified across all funders in the historical dataset that could be mapped onto each of the collated points of concern previously described. To facilitate this comparison, the five ‘ownership’ points of concern in Table 1 were further condensed into two overarching collated points of concern (‘ownership acquisition issues’ and ‘ownership husbandry/lifestyle issues’), producing a modified list with 27 entries. Other issues with inclusion criteria are noted in footnotes. Table 2 also includes comparative rankings across these 27 entries, calculated by total funding and by collated mean priority score, and is arranged by the collated mean priority score ranking. The top ten ranked highest priority collated points of concern all concerned issues related to various real-world aspects of human-canine interactions. Four concerned breeding and supply issues (including importation), three concerned issues related to canine behaviour, and three concerned issues related to clinical veterinary practice, such as the affordability and type of veterinary care.

**Table 2:** Highest priority topics from modified Delphi study that explored issues in canine health and welfare and its research funding, organised by problem category and compared with historical funding dataset from phase 1 of the overall research project.

ID number (used in subsequent data analysis)	Problem category	Specific issues	Overall (collated) mean priority score (lower number = higher priority)	Total funding in historical dataset	No. of grants in historical dataset	Priority rank	£ rank
1	4) Breeding and supply issues	Increasing the supply of healthy well-bred dogs.	1.00	£0.00	0	1	24
2	3) Societal issues	Dog bite attacks	1.00	£17,000.00	2	1	21
3	1) Canine behaviour	Human lifestyle and canine behaviour	1.00	£541,697.88	9	1	6
4	4) Breeding and supply issues	Uninformed puppy buying (HBC)	1.13	£54,800.00	4	4	20
5	7) Clinical practice	Access/affordability of veterinary care	1.14	£231,090.00	5	5	13
6	6) Issues related to importation	Welfare and importation	1.15	£201,662.00	3	6	15
7	4) Breeding and supply issues	Desire for extreme conformation	1.25	£225,293.08	6	7	14
8	7) Clinical practice	Veterinary overtreatment/euthanasia ^a^	1.25	£403,487.00	4	7	7
9	1) Canine behaviour	Poor understanding of canine behaviour	1.29	£330,352.00	5	9	9
10	7) Clinical practice	Welfare and real-world problems ^b^	1.33	£105,690.00	3	10	17
11	4) Breeding and supply issues	Online puppy trading	1.35	£196,662.00	2	11	16
12	7) Clinical practice	Common chronic disorders ^c^	1.35	£8,378,673.10	108	11	2
13	5) Breed-related diseases (overall)	Breed-related diseases ^d^	1.36	£11,146,494.50	277	13	1
14	3) Societal issues	Public ignorance about dogs	1.43	£305,835.00	4	14	10
15	2) Ownership issues	Ownership—lifestyle and husbandry	1.43	£382,638.15	11	14	8
16	2) Ownership issues	Ownership—acquisition issues	1.50	£291,282.00	8	16	12
17	6) Issues related to importation	Exotic diseases and importation ^e^	1.50	£2,120,663.00	6	16	3
18	3) Societal issues	Social media/popular culture	1.54	£0.00	0	18	24
19	4) Breeding and supply issues	Breeding regulation	1.57	£0.00	0	19	24
20	3) Societal issues	Cost of living crisis	1.57	£96,375.00	5	19	19
21	1) Canine behaviour	Canine behaviour	1.67	£1,797,760.21	20	21	4
22	4) Breeding and supply issues	Exploitative breeding for profit	1.84	£0.00	0	22	24
23	1) Canine behaviour	Training methods	1.92	£2,000.00	1	23	22
24	4) Breeding and supply issues	Which breeders produce healthier dogs	2.00	£2,000.00	1	24	22
25	7) Clinical practice	Long term health and medication/diet	2.00	£100,173.00	1	24	18
26	8) Shelter welfare	Long term shelter welfare	2.00	£299,540.22	7	24	11
27	4) Breeding and supply issues	Population and quantitative genetics ^f^	2.00	£1,406,165.25	88	24	5

The table includes comparative rankings calculated by total funding in historical dataset and by collated mean priority score and is arranged by collated mean priority score ranking.

^a^ All concerned euthanasia decisions.

^b^ Two heatstroke studies, one non-accidental injury study.

^c^ Includes funding that concerns multiple disorders in primary care practice, such as VetCompass.

^d^ Previous studies were deemed in scope if clinically relevant.

^e^ Includes SAVSNET funding, so not all directed towards exotic diseases.

^f^ Previous studies were deemed in scope if they centred dogs rather than having a multispecies remit.

The historical funding totals for each collated point of concern shown in Table 2 were also allocated by type of funder to differentiate grants supported by wide-scope funders (UKRI councils and the Wellcome Trust) from those supported by not-for-profit animal-directed funders. A detailed breakdown of this analysis is provided in the supplementary materials (‘Gap analysis with £’ sheet in S1 Dataset). This revealed that 20/27 (74.1%) of highest priority collated points of concern received no wide-scope funding at all during the period studied (2012–2022). Only one highest priority collated point of concern (long-term impact of medication and diet) received wide-scope funding but not animal-directed funding (a single Biotechnology and Biological Sciences Research Council (BBSRC) -funded study that explored the metabolic profiling of dogs with epilepsy to develop new nutritional treatments). Four highest priority collated points of concern received no direct specific funding from either wide-scope funders or not-for-profit animal-directed funders. These were: social media and popular culture; exploitative breeding for profit; increasing the supply of healthy well-bred dogs; and breeding regulation and enforcement. Table 3 provides a summary of the breakdown between funder categories at the problem category level, showing what percentage of total funding for each problem category originated from the animal-directed sector. Animal-directed funding accounted for the majority of funding in 6/8 problem categories, providing all the funding in 3/8 of them. The exceptions were clinical disease, where overall funding was roughly equal between funder categories, and issues associated with importation, where animal-directed funding provided just over a third of total funding. This was because SAVSNET funding included two large UKRI starting grants, which were mapped to this problem category.

**Table 3:** Highest priority problem categories from the modified Delphi study that explored issues in canine health and welfare and its research funding, organised by problem category and compared with historical funding dataset from phase 1 of research project, broken down by wide-scope versus animal-directed funder categories to show number of awarded grants and total funding awarded.

Problem category	No. of grants in dataset	Total relevant funding in dataset	No. of grants with wide-scope funding	Wide-scope funding	Wide-scope % of total category funding	No. of grants with animal-directed funding	Animal-directed funding	Animal-directed % of total category funding
1) Canine behaviour	35	£2,671,810.09	4	£959,705.00	35.9%	31	£1,712,105.09	64.1%
2) Ownership issues	19	£673,920.15	0	£0.00	0.0%	19	£673,920.15	100.0%
3) Societal issues	11	£419,210.00	0	£0.00	0.0%	11	£419,210.00	100.0%
4) Breeding and supply issues	101	£1,884,920.33	1	£133,989.00	7.1%	100	£1,750,931.33	92.9%
5) Breed-related diseases (overall)	277	£11,146,494.50	11	£3,391,829.56	30.4%	266	£7,754,664.94	69.6%
6) Issues related to importation	9	£2,322,325.00	2	£1,460,920.00	62.9%	7	£861,405.00	37.1%
7) Clinical practice	121	£9,219,113.10	22	£4,803,501.00	52.1%	99	£4,415,612.10	47.9%
8) Shelter welfare	7	£299,540.22	0	£0.00	0.0%	7	£299,540.22	100.0%

**Fig 3:**
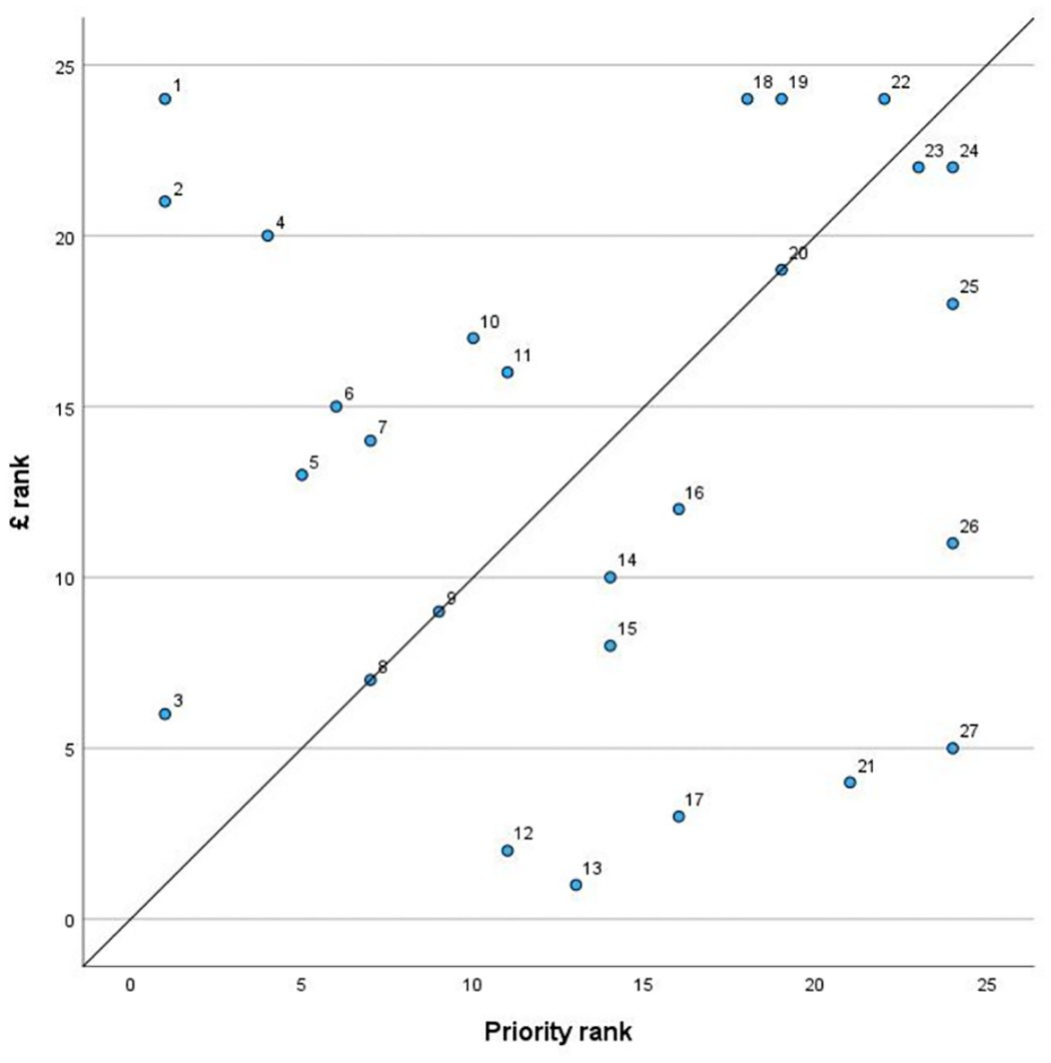
Ranked collated highest mean priority scores for points of concern for research topics in canine health and welfare obtained by modified Delphi study consensus, plotted against ranked total historical funding for each topic in previously obtained dataset. Diagonal line represents x = y, i.e. parity of ranking between these two measurements.

Associations between Delphi (collated) mean priority scores and past funding expenditure were explored to investigate relationships between consensus prioritisation and historical funding allocation, to develop a metric that identified which highest priority points of concern were relatively most ‘under’ or ‘over’ funded. Because historical funding allocation was very variable, a ranked system was used to create a more even comparative scale. Fig 3 depicts this comparison graphically by plotting priority ranking against funding ranking for the collated highest priority points of concern listed in Table 2 (ID numbers for data points in Fig 3 refer to Table 2). The diagonal line represents x = y, i.e. parity of ranking. Therefore, points on this line received the ‘expected’ funding for their priority score, while points above this line were relatively underfunded, with those furthest from the line receiving least funding relative to their priority score. Similarly, points below this line were relatively overfunded, with those furthest from the line receiving most funding relative to their priority score.

Table 4 provides a funding rank ratio index (FRRI) for each collated point of concern, produced by dividing the priority rank by the funding rank, in addition to the data used in Fig 3. With this FRRI metric, all values greater than one indicate relative underfunding, with the largest numbers indicating least funding relative to priority score. All specific issues that were relatively underfunded or with priority/funding parity concerned human-canine interactions in some way (as did some of the ‘overfunded’ issues). The two ‘most underfunded’ collated points of concern, by far, were ‘increasing the supply of healthy well-bred dogs’ (FRRI = 24.00) and ‘dog bite attacks’ (FRRI = 21.00), because these points of concern received little or no funding but were scored highest priority (1.00). The ‘impact of human lifestyle on canine behaviour’ also received a high FFRI (6.00), despite receiving significant historical funding, because of its highest prioritisation score (1.00), suggesting that even more funding was warranted for this research area. Other collated highest priority points of concern that received highest FFRI scores (≥2.00) were human behavioural change interventions in uninformed puppy buying (FFRI = 5.00); access/affordability of veterinary care (FFRI = 2.60); welfare relating to importation (FFRI = 2.50); and the human desire for extreme canine conformation (FFRI = 2.00). In contrast, some collated highest priority points of concern received much lower FFRI scores, reflecting considerable historical funding and relatively rather than inherently low prioritisation, since all these issues were pre-selected as highest priority. In some cases, these points of concern were broader and less specific than those with highest FFRI scores, which was reflected in their larger historical funding shares; for example, ‘common chronic diseases’ and ‘breed-related diseases’ had the lowest FFRI scores because of their very substantial overall previous funding.

**Table 4:** Funding rank ratio index for specific issues (highest priority collated points of concern) in canine health and welfare, calculated by dividing priority rank derived from Delphi study by funding rank derived from historical not-for-profit funding dataset (2012–2022).

ID number	Problem category	Specific issues (collated highest priority points of concern from Delphi study)	Overall (collated) mean priority score (lower number = higher priority)	Total funding in historical dataset	Priority rank	£ rank	Funding rank ratio index (priority rank/£ rank)
1	4) Breeding and supply issues	Increasing the supply of healthy well-bred dogs	1	£0.00	24	1	24.00
2	3) Societal issues	Dog bite attacks	1	£17,000.00	21	1	21.00
3	1) Canine behaviour	Human lifestyle and canine behaviour	1	£541,697.88	6	1	6.00
4	4) Breeding and supply issues	Uninformed puppy buying (HBC)	1.13	£54,800.00	20	4	5.00
5	7) Clinical practice	Access/affordability of veterinary care	1.14	£231,090.00	13	5	2.60
6	6) Issues related to importation	Welfare and importation	1.15	£201,662.00	15	6	2.50
7	4) Breeding and supply issues	Desire for extreme conformation	1.25	£225,293.08	14	7	2.00
10	7) Clinical practice	Welfare and real-world problems	1.33	£105,690.00	17	10	1.70
11	4) Breeding and supply issues	Online puppy trading	1.35	£196,662.00	16	11	1.45
18	3) Societal issues	Social media/popular culture	1.54	£0.00	24	18	1.33
19	4) Breeding and supply issues	Breeding regulation	1.57	£0.00	24	19	1.26
22	4) Breeding and supply issues	Exploitative breeding for profit	1.84	£0.00	24	22	1.09
8	7) Clinical practice	Veterinary overtreatment/euthanasia	1.25	£403,487.00	7	7	1.00
9	1) Canine behaviour	Poor understanding of canine behaviour	1.29	£330,352.00	9	9	1.00
20	3) Societal issues	Cost of living crisis	1.57	£96,375.00	19	19	1.00
23	1) Canine behaviour	Training methods	1.92	£2,000.00	22	23	0.96
24	4) Breeding and supply issues	Which breeders produce healthier dogs	2	£2,000.00	22	24	0.92
16	2) Ownership issues	Ownership—acquisition issues	1.5	£291,282.00	12	16	0.75
25	7) Clinical practice	Long term health and medication/diet	2	£100,173.00	18	24	0.75
14	3) Societal issues	Public ignorance about dogs	1.43	£305,835.00	10	14	0.71
15	2) Ownership issues	Ownership—lifestyle and husbandry	1.43	£382,638.15	8	14	0.57
26	8) Shelter welfare	Long term shelter welfare	2	£299,540.22	11	24	0.46
27	4) Breeding and supply issues	Population and quantitative genetics	2	£1,406,165.25	5	24	0.21
21	1) Canine behaviour	Canine behaviour	1.67	£1,797,760.21	4	21	0.19
17	6) Issues related to importation	Exotic diseases and importation	1.5	£2,120,663.00	3	16	0.19
12	7) Clinical practice	Common chronic disorders	1.35	£8,378,673.10	2	11	0.18
13	5) Breed-related diseases (overall)	Breed-related diseases	1.36	£11,146,494.50	1	13	0.08

Higher funding rank ratio index values indicate ‘more underfunded’, i.e. greater indication for future increased funding.

### Gap analysis of common chronic conditions in primary care practice

As described earlier, the results from a previous VetCompass study were used to create a list of common chronic conditions in primary care practice together with their prevalence values [24]. That full study reported 29 individual level common disorders that had an annual diagnosis prevalence > 1%. This list was filtered to remove inherently acute or heterogenous conditions. Among the remaining conditions, all non-mass skin disorders were collated and their prevalence summed, to develop a category that could be compared with the historic data. The same process was used to collate common undesirable behaviours. ‘Heart murmur’ was mapped onto ‘heart murmur and mitral valve disease (MVD)’ in the historic data, because MVD, while the commonest cause of heart murmur in canine practice, is often not specifically recorded as a diagnosis in primary care records, yet MVD as a term is often identified as the subject of health research [25]. This cleaning and collating process yielded a final list of ten common disorders with a prevalence > 1%, which were considered as chronic and specific (Table 5).

**Table 5:** A) Individual 29 most prevalent (>1%) disorders and B) extracted 10 most common chronic collated disorders in primary canine veterinary care.

A) Data transferred from O’Neill et al (2021) [38,39] (see legend)			B) Data from A, collated to list common chronic disorders	
Individual disorder term used in original VetCompass study	Prevalence (%) in original VetCompass study	Accepted as chronic/recurring	Collated chronic disorder term used in the current study	Prevalence (%) used for the current study
Periodontal disease	12.52	Yes	Periodontal disease	12.52
Otitis externa	7.30	Yes	Otitis externa	7.30
Obesity	7.07	Yes	Obesity	7.07
Overgrown nails	5.52	Yes	Skin disorders (non-mass)	5.81
Anal sac impaction	4.80	Yes	Overgrown nails	5.52
Osteoarthritis	2.34	Yes	Anal sac impaction	4.80
Aggression	2.24	Yes	Behavioural disorders	3.74
Heart murmur	2.13	Yes	Osteoarthritis	2.34
Pruritus	1.63	Yes	Heart murmur	2.13
Allergy	1.57	Yes	Patellar luxation	1.04
Undesirable behaviour	1.50	Yes		
Pyoderma	1.46	Yes		
Atopic dermatitis	1.15	Yes		
Patellar luxation	1.04	Yes		
Diarrhoea	3.81	No		
Vomiting	3.04	No		
Lameness	2.65	No		
Conjunctivitis	2.24	No		
Skin mass	2.07	No		
Flea infestation	2.05	No		
Lipoma	1.44	No		
Claw injury	1.38	No		
Pododermatitis	1.36	No		
Gastroenteritis	1.33	No		
Foreign body	1.27	No		
Post-operative wound complication	1.19	No		
Wound	1.12	No		
Skin cyst	1.10	No		
Retained deciduous tooth	1.01	No		

Data taken from O’Neill DG, James H, Brodbelt DC, Church DB, Pegram C. Prevalence of commonly diagnosed disorders in UK dogs under primary veterinary care: results and applications. BMC Veterinary Research. 2021;17(1):69.

The historical dataset created during the first phase of the current project was interrogated to extract the information on how much research funding had been directed towards each of these ten most prevalent common chronic disorders, as discussed earlier and shown in Table 6 [5]. This revealed that £4,525,023.96 of funding was directed towards these ten disorders in total, including both support from wide-scope funders (UKRI councils and the Wellcome Trust) and animal-directed funders. Wide-scope funders contributed £2,277, 725.00 (50.34%) of this funding, and animal-directed funders contributed £2,247,298.96 (49.66%). However, the proportion of funding provided by each type of funder varied greatly between disorders, as shown in Table 6.

**Table 6:** Ten most prevalent chronic collated disorders recorded in dogs under primary veterinary care in the UK (O’Neill et al, 2021) [38,39] mapped onto historical UK not-for profit canine research funding data to determine proportional actual and prevalence-based ‘fair share’ of total funding and funding from wide-scope and animal-directed funders per disorder.

A: Condition	B: Prevalence % (VetCompass data)	C: Total funding of topic (wide-scope and animal-directed funders)	D: Total wide-scope funding of topic	E: Total animal-directed funding of topic	F: Animal-directed funding as % of total	G: Total prevalence-based proportional spend ("fair share")^a^	H: % of total “fair share” actually spent (100% represents a fair share)^b^	I: Wide-scope prevalence-based proportional spend (“fair share”)^c^	J: % of wide-scope “fair share” actually spent (100% represents a fair share)^d^	K: Animal-directed prevalence-based proportional spend (“fair share”)^e^	L: % of animal directed “fair share” actually spent (100% represents a fair share)^f^
Periodontal disease	**12.52**	£38,860.00	£0.00	£38,860.00	100.00%	£1,083,858.81	**3.59%**	£545,614.52	**0.00%**	£538,244.28	**7.22%**
Otitis externa	**7.30**	£100,011.00	£0.00	£100,011.00	100.00%	£631,962.40	**15.83%**	£318,129.87	**0.00%**	£313,832.53	**31.87%**
Obesity	**7.07**	£761,335.00	£415,141.00	£346,194.00	45.47%	£612,051.26	**124.39%**	£308,106.60	**134.74%**	£303,944.66	**113.90%**
Skin disorders (non-mass)	**5.81**	£355,029.71	£101,520.00	£253,509.71	71.41%	£502,972.82	**70.59%**	£253,196.52	**40.10%**	£249,776.30	**101.49%**
Overgrown nails	**5.52**	£0.00	£0.00	£0.00	n/a	£477,867.46	**0.00%**	£240,558.48	**0.00%**	£237,308.98	**0.00%**
Anal sac disease	**4.80**	£18,000.00	£0.00	£18,000.00	100.00%	£415,536.92	**4.33%**	£209,181.29	**0.00%**	£206,355.64	**8.72%**
Behavioural disorders	**3.74**	£1,317,212.99	£631,221.00	£685,991.99	52.08%	£323,772.52	**406.83%**	£162,987.09	**387.28%**	£160,785.43	**426.65%**
Osteoarthritis	**2.34**	£1,446,532.66	£1,129,843.00	£316,689.66	21.89%	£202,574.25	**714.08%**	£101,975.88	**1107.95%**	£100,598.37	**314.81%**
Heart murmur/MVD	**2.13**	£488,042.60	£0.00	£488,042.60	100.00%	£184,394.51	**264.67%**	£92,824.20	**0.00%**	£91,570.31	**532.97%**
Patellar luxation	**1.04**	£0.00	£0.00	£0.00	n/a	£90,033.00	**0.00%**	£45,322.61	**0.00%**	£44,710.39	**0.00%**
**Total**		**£4,525,023.96**	**£2,277,725.00** ^g^	**£2,247,298.96** ^h^		**£4,525,023.96**		**£2,277,897.06** ^g^		**£2,247,126.90** ^h^	

^a^ Calculated as (∑ C /∑ B) = £86,570.19 x B for each condition.

^b^ Calculated as (C/G x 100%) for each condition.

^c^ Calculated as 50.34% of total “fair share” for each condition.

^d^ Calculated as (D/I x 100%) for each condition.

^e^ Calculated as 49.66% of total “fair share” for each condition.

^f^ Calculated as (E/K x100%) for each condition.

^g^ and ^h^–paired figures differ slightly due to rounding errors.

This analysis revealed clear funding ‘winners’ and ‘losers’ among the common chronic disorders. Three conditions received more than twice their ‘fair share’ of total funding. Osteoarthritis and behavioural disorders were heavily funded by both wide-scope and animal-directed funders, with osteoarthritis receiving more than 10x its ‘fair share’ of wide-scope funding. Heart murmur/mitral valve disease received over 5x its ‘fair share’ of animal-directed funding, but was not supported by wide-scope funders. Obesity and skin disorders both received roughly their expected ‘fair share’ of total funding (measured as between 50% and 200% of their ‘fair share’), and were both supported by wide-scope and animal-directed funders. The other five conditions all received much less total funding than their ‘fair share’; and none of these received any wide-scope funding. Otitis externa received roughly a third of its ‘fair share’ of animal-directed funding. Periodontal disease and anal sac problems were relatively severely underfunded, each receiving less than a tenth of their ‘fair share’ of animal-directed funding; moreover, the £18K of anal sac disorder funding in the historical dataset concerned anal adenocarcinoma rather than anal sac impaction, which received no funding. Overgrown nails and patellar luxation received no funding at all within the study dataset.

#### 3.6.2. Research approaches and methodologies

As previously described, 23 highest priority points of concern from the Delphi study which concerned research approaches and methodologies were taken forward for analysis. One point, ‘research that informs human medicine’, priority score 2.0, was excluded from further analysis at this stage, because the Delphi participants had agreed that research primarily intended to advance human medicine was outside the remit of the animal-directed funding sector. The remaining points were examined for overlap and collated where appropriate, creating overall mean priority scores for collated points as described previously. New problem categories of research design, investigative approach and research engagement emerged during this process, creating a summarised list of 7 specific issues that concerned highest priority approaches and methodologies for future research, as shown in Table 7. The three highest priority collated points of concern in this category all related to designing research with maximal effectiveness for improving canine lives. These were: embedded human behavioural change interventions; built-in project impact; and focus on welfare in research design.

**Table 7:** Delphi consensus highest priority approaches and methodologies for future research in canine health and welfare.

Problem category	Original discussion points	Type of problem	Specific issues	Comments from participants, summarised	Overall (collated) mean priority score (lower values reflect higher priority)
Research design	Collation of one point from C: testing of possible interventions to assess HBC effectiveness (1.25) and one point from D: built-in linking of clinical research to HBC research to aid implementation, where appropriate (1.29).	HBC inclusion	Embedding and testing human behavioural change interventions	Generally agreed that embedding and testing HBC interventions is crucial where appropriate, including specialist HBC input where needed—but some research doesn’t need it.	1.27
	Collation of two points from B: funding overlooks real world impact/implementation (1.40) and pathway to impact not built into project design (2.00); one point from C: translating research findings into practical actions (1.0); and two points from D: prioritising problems with more impact (1.33), and more focus on impact (1.57).	Research with impact	Need to prioritise projects with practical impact and build it into project design	Some caution that outcomes are not always anticipated at the start of research, so excessive focus on impact can be counterproductive, but overall prioritising impact for dogs in research design was considered very high priority.	1.46
	Collation of three points from C: reasons for neglect/poor welfare (1.00); investigative approach that focuses on welfare (1.00); and improving/enforcing welfare legislation (2.00); and one point from D: more research that investigates underlying reasons for welfare issues (1.43).	Broad focus of research	Focus on welfare in research design	Generally agreed very high fundamental priority. Some emphasis on specifically investigating legislative efficacy.	1.36
	D		Research designed to fit needs of sector	Generally agreed research should fit the needs of dogs, but some discussion about what this means in reality.	2.00
Investigative approach	Collation of one point from B: funding misdirected, overlooks social science/HBC research (1.25); one point from C: anthrozoology of dog ownership (1.33); and two points from D: more social science/applied research (1.67) and more humanities research (2.00)	Humanities and social science approaches (HSS)	More HSS research: a) social science b) humanities c) anthrozoology	Generally all considered high value and often overlooked (some score reduction from people who were unaware of these approaches, arguably demonstrating that they are sometimes overlooked!)	1.56
	Collation of 3 points from B and C, which are described in the ‘specific issues’ column; all with priority score of 2.00	Specific types of clinical study	a) Not enough prospective studies b) Not enough randomised controlled trials c) Not enough studies on complex issues	Generally felt more studies of these types would be useful, but various factors besides funding limit their deployment.	2.00
Research engagement	Collation of one point from A: obstinacy/polarised views/reluctance to change (1.67); one point from C: more owner/vet engagement (1.75); and one point from D: more effective communication to owners and sector professionals (1.86).	Research outreach communication	Ineffective communication, barriers to change	Emphasis on effectiveness of communication outreach to different groups to ensure public engagement with research outputs	1.76

#### 3.6.3. Research processes and research funding infrastructure

As described earlier, 33 highest priority points of concern from the Delphi workshop which concerned research processes and infrastructure were taken forward for analysis. One point, ‘hard to recruit numbers for valid study’, priority score 1.75, was excluded from further analysis at this stage, because it concerned a specific difficulty with the execution of research, whereas all the other highest priority points in this section concerned research processes and funding infrastructure, and thus contributed to an overview of suggested sector reforms. The remaining 32 points were examined for overlap and collated where appropriate, creating overall mean priority scores for collated points as described previously. New problem categories of overarching issues, funding processes and researcher issues emerged during this process, creating a summarised list of 15 specific highest priority issues, as shown in Table 8. The highest priority collated point of concern was the overall lack of funding in this sector (priority score 1.00), which was scored much more highly than the second highest priority collated point of concern in this category (more support for early career researchers, priority score 1.48). Overall, there was a strong emphasis on the need for increased transparency and collaboration in this sector, with 8/15 highest priority collated points of concern addressing this subject.

**Table 8:** Delphi consensus highest priorities for future change in research processes and research funding infrastructure for future research in canine health and welfare.

Problem category	Original discussion points	Specific issues	Comments from participants, summarised	Overall (collated) mean priority score (lower values reflect higher priority)
1) Overarching issues	Collation of one point from B: overall lack of funding/too few funders (1.00) and one from D: more money! (1.00).	Overall lack of funding/too few funders	A universal and unsolvable problem, which certainly does significantly limit the scope and quantity of canine relevant research.	1.00
	Collation of one point from B: overarching issue of gaps/barriers between sectors—charity/veterinary/academic/breeders (1.60); and two points from D: linking academia to frontline sector to improve relevance of research (1.43), and collaboration between sectors (industry/charity/pharma, etc) (2.00).	Reducing gaps and barriers, increasing overall collaboration and communication between sectors.	Includes planning research with real-world factors in mind where needed, overcoming misconceptions and barriers between sectors which impede research to improve canine welfare (e.g. disconnects and lack of visibility between various stakeholder groups).	1.68
	Collation of one point from B: government/wide-scope funders overlook/exclude canine sector (1.75) and one point from D: more government funding/political prioritisation (2.00)	Canine sector not prioritised by government and wide scope funders	Generally agreed that this is true and has various causes. Variation in whether participants thought this was inevitable or could be changed.	1.88
	B	Largest (UKRI and similar) grants usually restricted to One Health/public health	Generally agreed that this is true, that it limits what is funded and that it drives canine researchers to spin proposals to fit this agenda, which can be detrimental.	2.00
2) Funding processes	D	More transparent and accessible industry funding	Agreed that more accessible funding is always a good thing. Definition of industry can be very broad—includes any non-charitable business; not all such funding is advertised openly, so improving access could be useful.	1.71
	Collation of one point from B: centralised coordination of strategic priorities (1.40); and two points from D: collaborative centralised identification of priorities and funding gaps (1.86), and more long-term strategic planning to prioritise work most likely to improve welfare (2.00).	Collaborative centralised discussion of priorities and funding gaps to develop overall strategic plans for future funding	Generally agreed that this would be very helpful in theory to improve efficiency for all parties and maximise welfare value of research, but with caveats that it would need to be hosted independently to avoid giving power to one contributor, that it might be difficult to implement, and that planning cannot foresee or account for all eventualities.	1.75
	Collation of one point from C: ‘big’ investment into any major area of canine health, e.g. cancer, pain (2.00); and two points from D: more larger/collaborative grants (allowing broader scope/higher quality studies (2.00), and collaboration between funders, to support larger projects (1.43).	Collaboration between funders to support ’big’ projects with greater scope and impact.	Generally considered a good thing, but caveats that it may divert funds from worthwhile smaller projects and that the politics or logistics of collaboration may be difficult.	1.81
	Collation of one point from B: lack of centralised opportunities for collaboration (2.00); and one point from D: clearer visibility of opportunities for collaboration (1.71).	Centralised visibility of future opportunities for collaboration, for funders AND researchers	Generally agreed that this is lacking and would be very helpful if it could be implemented. No clear vision of preferred platform/infrastructure, however.	1.86
	Collation of two points from D: better visibility of current funding patterns (2.00) and collaborative centralised live database of funded projects and their progress/outputs (2.00).	Better visibility of past funding patterns, ideally through collaborative centralised live database	Consensus that public visibility of funding patterns currently varies enormously between funders, and that improving this would be useful, especially to researchers. However, concerns re cost, logistical and political feasibility. N.B., UKRI councils do this already.	2.00
	D	Collaborations between research centres investigating same topic, facilitating larger or ’better powered’ projects	Generally agreed that intellectual collaboration between research centres is positive and can advance canine health and welfare, although some caveats that it can be politically or logistically difficult.	1.86
	C	More funding for start-ups/pilots to lever sector change	Multiple views here—some thought this was high priority to encourage innovation, some thought this was low priority on the basis that pilot projects seldom seed bigger studies.	2.00
3) Researcher issues	Collation of two points of concern from B: not enough support/funding for PhD and ECR roles (1.25) and lack of career structure, ECRs marginalised (1.25); and two points from D: more support for ECRs (1.43) and built-in ECR support within bids (2.00).	More support for early career researchers	Agreed that ECR career structure is a massive issue, particularly post-PhD, where much talent is lost because of precarity, little funding and poor remuneration. Arguably therefore also a problem for canine welfare. Funding and mentorship within bids/grants would help.	1.48
	Collation of one point from B: grant writing takes much time, extra to main (heavy) workload (1.75); and one point from D: ability to submit same grant application to multiple funding bodies (1.57).	Burden of writing individual grants for each funder	Researchers felt very strongly that writing separate applications for each funder is a huge time burden which impacts both human and canine welfare. They would welcome any level of simplification of this process: a common online portal for preliminary applications, a common preliminary application form, or even just a common agreed format for CVs. Some funders did not want to see proposals that had been sent elsewhere.	1.66
	Collation of two points of concern from B: accessibility as career path, e.g. if part time/employed (1.50) and networking/personal contacts too important, process mysterious for outsiders (1.75); and one point from D: more outreach to researchers/opportunities to connect (2.00).	Networking, accessibility and outreach initiatives	Agreed that barriers to entry to research, difficulty of establishing a network, etc., reduce diversity and lose talent, which potentially impacts canine welfare. Funder initiatives could improve outreach and encourage wider participation—but also caution that this could ’poach’ research ideas or cause false hope in those not later funded.	1.75
	Collation of one point from B: useful feedback not given if proposal rejected (2.00) and one point from D: better feedback (1.57).	Better feedback if grant proposal rejected	Agreed by researchers that this is a big issue because they don’t know how to improve proposals, thus also potentially impacts canine welfare; also not transparent. Funders argue no time to provide individual feedback.	1.79

## 4. Discussion

This study deployed a modified Delphi approach to establish group consensus about the highest priority topics in UK canine and welfare and its research; to identify the highest priority future research approaches and methodologies; and to identify highest priorities for future reform in research processes and infrastructure.

Collated highest priority points of concern that were classified as relating to research topics in canine health and welfare were inductively grouped within eight problem categories: canine behaviour, ownership issues, societal issues, breeding and supply issues, breed-related diseases, issues related to importation, clinical practice and shelter welfare. Notably, almost all these 30 highest priority collated points of concern addressed various aspects of the human-canine relationship, either considering human decisions that affect canine lives (for example, breeding and supply issues) or canine problems that also affect people (such as dog bite attacks or exotic diseases linked to importation). Only two highest priority points in this group—common chronic diseases and the impact of medication and diet on canine health—could be described as primarily canine clinical problems with little human factor involvement (and arguably, even problems in these categories may be influenced by human behaviours: obesity, for example). This finding emphasises the importance of human-canine interactions to the welfare of both dogs and people, confirming that understanding human behaviour is a key element of canine health and welfare research [26,27].

Notably, the finding of frequent human factor involvement in the highest priority points of concern was not an *a priori* expectation of this study. As discussed later, well over half of the participants were veterinary professionals and/or research scientists, and the points of concern that were discussed at the workshop had all been suggested by the participants themselves, following the Delphi approach. Consequently, physical disease might have been expected to feature more strongly in the highest prioritisation data than it did. Although 9/27 highest priority issues in Table 2 do relate to physical disease, most of these points (such as ‘which breeders produce healthier dogs’) concern physical health issues framed in terms of human activity and choices, rather than as entirely clinical phenomena, even though there were several participants in every discussion group with deep clinical expertise. As discussed later, the finding of high prioritisation of human factors will inevitably have been culturally influenced by the experiences of these UK participants, and the balance of priorities might have varied in other countries. Nevertheless, this insight confirms that those research funders who direct resources towards investigating human factors in canine wellbeing or in driving human behavioural change are responding to a widely perceived highest priority need in this sector in the UK.

Moreover, discussions between the Delphi participants repeatedly emphasised the complexities of the human-canine relationship. For example, general agreement was frequently expressed that the various issues linked with ownership and breeding are highly interconnected; to quote one participant, ‘*I think it’s all tied up with the same thing about poor understanding of dog*s*’ needs*, *expecting them to fit around our needs completely*, *and a supply and demand issue linked to instant gratification and getting a dog that looks right*.*’* Where points of concern addressed issues in canine welfare that were currently topical, comments frequently revealed deep understanding and widespread awareness of these problems among the participants, who tended to score such issues as higher priority. For example, issues related to importation, conformation-related disease and the cost-of-living crisis were all rated highest priority. All these were topical in the UK at the time of the workshop: however, conformation-related disease has been a consistent major concern for over a decade, whereas the cost-of-living crisis was frequently described in workshop transcripts as a recent change [28]. In a few cases, prioritisation scores were perhaps more surprising. There was widespread pushback against the original point of concern ‘breeding for money’ in part A (which was consequently reworded), with most groups agreeing that, while exploitative breeding for profit was ‘*the dark end of dog breeding*’, there was nevertheless a need for ethically bred puppies to be produced in sufficient numbers to satisfy consumer demand, and that therefore responsible high welfare commercial breeding should perhaps be supported more; ‘*I don’t have a problem with it*, *and I never would have thought I’d have said that*’(voiced by a charity worker). Similarly, in part C, ‘how to increase the supply of healthy well-bred dogs’ was rated highest priority by all groups that scored this point, with almost no discussion deemed necessary to come to this consensus. On this subject, the Delphi format arguably thus captured perspectives that might not have been anticipated and which may reflect highly topical changes in stakeholders’ positionalities, with the overall consensus supporting the need for ethical dog breeding, despite the inclusion of charity workers and veterinary professionals in every discussion group who might have been expected to hold different views.

The comparison of the highest priority research topics with previous funding for these topics in the historical dataset revealed some interesting insights [5]. Animal-directed funders supported a much wider range of highest-priority research topics than wide-scope funders, although the individual grants awarded by wide-scope funders tended to be larger. Wide-scope funding of high priority topics tended to fall into two broad categories. There was substantial wide-scope funding for several clinically relevant topics, much of which came from BBSRC grants for large-scale projects framed in a One Health or multi-species context. Perhaps more surprisingly, wide-scope funders also provided several large grants to investigate canine-relevant veterinary issues, such as the ethics of companion animal euthanasia, from a humanities perspective. Animal-directed funders dominated in problem categories related to more practical aspects of the human-canine relationship, such as ownership issues and issues related to breeding practices. Surprisingly, however, no specific historical funding at all was identified for several highest priority topics, such as the role of social media/popular culture in canine welfare and exploitative breeding for profit (criminology, etc), although some previously funded research did provide some contextual insights into such topics. These topics may have been less researched because they have only recently been recognised (as in the example of the role of social media), are hard to define, difficult or even dangerous to investigate, or require a complex methodological approach. Funding bodies’ areas of remit, limited resources and prioritisation decisions also inevitably mean that some potentially valuable topics go unfunded or ‘under’ funded, as discussed further below.

Highest priority topic rankings were plotted against previous funding ranking and compared through a funding rank ratio index to assess relative ‘under’ and ‘over’ funding in the historical funding allocation dataset. As shown in Table 4, the ‘most underfunded’ topics all concerned real-world issues relating to canine welfare. Since this table only included topics that were rated highest priority during the Delphi study, all these topics were by definition considered important by stakeholder consensus (which included multiple people who make funding decisions) at the time of the workshop. These topics may have previously been relatively neglected because they are potentially challenging to investigate, because they are genuinely new problems (for example, exotic diseases related to importation in the UK), or because rather than actually being new issues, they are currently in the process of being identified as matters that need attention as stakeholders’ understandings evolve (for example, the top ‘most underfunded’ topic, ‘increasing the supply of healthy well-bred dogs’, discussed previously above). Moreover, not all funders are willing to finance research into real-world canine issues relating to canine welfare. Our previous analysis of historical funding data revealed that most wide-scope funders are unlikely to support canine-focused research projects, and that some animal-directed funders primarily support clinical topics, so that UK-funded research into real-world issues relating to canine welfare is largely dependent on support from a small number of animal-directed funders, despite its high prioritisation in the current study [5]. Since these animal-directed funders must decide how to divide limited resources across the whole spectrum of canine-focused research topics, potentially also including all types of clinical disease, the high prioritisation of human factor real-world issues is an important finding from the current study that may usefully inform these difficult decisions. Funders who are concerned about assessing the relative merits of research proposals could use standardised tools, such as the ‘benefit for the dog’ and ‘pathway to impact’ metrics presented in our previous paper from the current research project [5], to overcome any difficulties and biases that hamper the comparative evaluation of research proposals across a wide range of potentially important topics, thus better ensuring that their resources are distributed to most advantage.

The main areas of attention indicated for increased research funding from the current work are clearly the breeding and supply of dogs. Of the twelve ‘most underfunded’ topics with a funding rank ratio > 1 (i.e., above parity), six were in this problem category: increasing the supply of healthy well-bred dogs; addressing uninformed puppy buying through human behavioural change; the human desire for extreme conformation; online puppy trading; breeding regulation; and exploitative breeding for profit. Two other ‘most underfunded’ topics, namely welfare issues related to importation and social media/popular culture, also concern related subjects. Of the remaining four ‘most underfunded’ topics, two (dog bite attacks and access/affordability of veterinary care), were highly topical at the time of the workshop in autumn 2023. The participants revealed their subject knowledge in rating dog bite attacks as a highest priority topic (score 1.0), just a few weeks before the UK Government introduced new legislation to control the breeding, sale and ownership of XL Bullies—a possible measure mentioned in some workshop discussions [29]. The cost of veterinary care was also a major current issue in the UK at the time of the workshop, as shown by the expected subsequent publication of the UK’s Competition Markets Authority’s report on veterinary pricing [30]. The lack of past research funding on these topics in the historical dataset (which included research funded as recently as the end of 2022) in part reflects the inevitable time lag between the need for research and its execution. However, the prioritisation of these topics within the ‘most underfunded’ quadrant reveals their topical importance within the canine sector at the time of this study, some weeks before these issues reached the mainstream media.

The final topics in the ‘most underfunded’ category were the impact of human lifestyles on canine behaviour, which was flagged by this metric because of its highest prioritisation, despite having attracted more historical funding than any of the other ‘most underfunded’ points of concern, and specific real-world problems that cause welfare issues. The illustrative example provided by the Delphi participant who suggested this point of concern was the issue of paralysed dogs in carts. There was no funded research on this specific subject in the historical dataset, but previously funded research into heatstroke disorder and non-accidental injury were mapped onto this topic as comparable real-world issues. Issues of this kind, which arise from specific real-world external circumstances and are not necessarily directly related to fields of clinical specialism, could easily be overlooked by researchers and funders, although arguably there is a great need for good evidence to inform interventions with problems of this type, since by definition they impact welfare. Therefore, there is considerable potential value for canine welfare in highlighting this ‘most underfunded’ topic as a possible area for future attention.

However, the identification of underfunded research topics in the current study certainly had methodological limitations. In particular, the historical funding dataset was confined to identified UK not-for-profit funders, excluding any in-scope funders that were not identified, international research, UK research with international funding, research funded by corporate practices, commercial funding and any research carried out internally by salaried staff in any organisation [5]. Moreover, specifically identified research topics in the historical dataset were confined to those that could be individually ascertained from project descriptions. Consequently, the historical dataset inevitably excludes many possibly relevant research projects. For example, as noted above, the current analysis identified dog bite attacks as a highest priority yet relatively underfunded topic, only addressed by two research grants in the historical dataset. One of these was an Economic and Social Research Council PhD award to the University of Stirling, listed in the UKRI database but with no linked specific funding award; the other was a £17,000 award from an animal-directed funder to Professor Carri Westgarth at the University of Liverpool. Yet a substantial volume of research into dog bite attacks has been carried out at Liverpool, by Westgarth’s research group and by Dr John Tulloch, supported by grants with other primary aims or funded by other means, and hence excluded from this historical dataset [31–34]. Some conditions that were studied within larger umbrella projects will have been omitted from the dataset because they were not individually identified by specific research grants: for example, the VetCompass and SAVSNET epidemiological surveillance projects have produced many papers that investigate specific clinical problems in dogs and other species, but which were funded by general umbrella grants, so that this funding could not be precisely mapped to all destination topics that these projects address [35,36]. Historical research topic funding may thus well be under-recorded within the project dataset. Rarely, it may also have been over-recorded, when umbrella grants were mapped onto relevant research topics that certainly accounted for some, but potentially not all, of the recorded expenditure. Therefore, the findings in the current study should be interpreted with care, considering other funding sources and contextual factors. It would have been impractical to address this limitation by conducting a full literature review to identify out-of-scope research for every topic discussed in the current paper. However, where the current study flags a topic as relatively underfunded, it would be wise to conduct a supplementary literature search to identify any out-of-scope research before assuming that this inference is definitive.

The detailed comparative analysis of previous funding of ‘common chronic’ disorders provided a novel way of surveying the distribution of research resources across a wide range of conditions which, because of their high prevalence and chronicity in primary care practice, have substantial impact on canine welfare [4]. The current analysis did not consider the relative severity of these conditions and made some other methodological approximations (such as mapping heart murmurs onto MVD). While it could be argued that overgrown nails, which received no research funding at all, are a clinically simple problem to address, it is perhaps more surprising that there was no funded research on patellar luxation in the historical dataset, despite it being a commonly reported cause of lameness that is both poorly understood and increasing in prevalence [37]. Anal sac disease and periodontal disease are also very common conditions that received surprisingly little research attention in this dataset [38,39]. Periodontal disease has long been recognised as the ‘most commonly diagnosed … and most undertreated’ companion animal health problem, is known to be a painful condition that has considerable impact on systemic health and wellbeing, and had the highest prevalence in the reference study used here, yet received only 3.59% of its total ‘fair share’ of research funding [40]. This disorder is therefore a strong candidate for further clinical research aimed at maximising welfare outcomes.

The 22 highest priority Delphi points of concern that concerned research approaches and methodologies were collated into three problem categories, highlighting areas for future change. The first category, research design, included a focus on human behavioural change, where appropriate; research designed with impact in mind; research focused on welfare; and research designed to fit the needs of the sector. The second category, investigative approaches, included two types of approach that were scored as highest priority for future research: projects with a humanities and/or social science perspective; and various specific types of clinical study which were considered under-utilised: prospective studies, randomised controlled trials and studies on complex issues (it was recognised that practical and financial constraints often restrict this type of investigation). The final category to emerge from this section of the analysis was research engagement: participants agreed that embedded public engagement with relevant stakeholders to communicate research outputs and thus increase their impact was a high priority for future projects, where appropriate. Overall, therefore, participants strongly prioritised real-world considerations in the design and execution of canine health and welfare research and had a strong focus on research that engaged with the human factors in canine welfare, apart from the points that concerned specific clinical topics.

It was generally impractical to compare the consensus highest priorities in research approaches and methodologies with the historical dataset, because there was often insufficient information to analyse the historical data in terms of these parameters. For example, it was impossible to establish if past projects had considered human behavioural change interventions or routes to public engagement without access to either the full research proposal or any published outputs, and for many grants neither was available. However, the previous paper from the current research project does evaluate the historical dataset in terms of a customised pathway to impact metric, although that considered effective impact rather than exploring whether impact was explicitly built into project design [5].

Finally, 32 Delphi consensus highest priorities for future change in research processes and research funding infrastructure were condensed into three problem categories: overarching issues, issues with funding processes and researcher-facing issues, with 15 collated points of concern between them. One aim of this section was to provide insight for funders, to inform their future research processes. Three of the four points grouped within overarching issues (overall lack of funding; canine sector not prioritised by wide scope funders; largest grants usually restricted to One Health/public health), while considered highest priority, did not concern matters within the control of animal-directed funders, and thus cannot serve as action points emerging from this study. The fourth overarching issue, the importance of reducing gaps and barriers to increase overall collaboration and communication between sectors, again reflects the emphasis on real-world impact and human factors in canine health and welfare that was a recurring theme throughout this Delphi study.

The second problem category within the section on research funding infrastructure and processes concerned highest priority issues with funding processes. Here, there was a strong consensus opinion on the importance of collaboration and transparency, which were addressed by 5/7 collated points in this category. These suggested reforms were: collaborative centralised strategic discussion of funding gaps and future funding priorities and funding gaps; collaboration between funders and between researchers investigating the same topic, in both cases to enable projects with greater scope; and better visibility of past funding patterns and of future opportunities for collaboration, for funders AND researchers, ideally through a collaborative centralised live database. The remaining two points in this category were the provision of more transparent and accessible industry funding and of more funding for start-up and/or pilot research projects to lever sector change. Overall, therefore, Delphi participants strongly supported a shift towards more collaborative funding processes, albeit with some caveats and nuances in the discussion, which are captured in the transcription summaries.

The final problem category within the section on research funding infrastructure and processes concerned highest priority issues for researchers. The four points in this category were split between issues for junior and senior researchers. Participants felt strongly that there should be greater structured support for early career researchers, both in terms of more specific provision within grant arrangements (particularly provision of salary support for postdoctoral researchers) and in terms of providing networking, accessibility and outreach initiatives. They noted that the barriers to accessing a research career, to career stability and to progression not only cause practical difficulties for those trying to follow a career in research, particularly for people from non-traditional research backgrounds, but also, through the consequent ongoing attrition of trained researchers from this sector, impacts the efficacity of research in advancing canine health and welfare, although they acknowledged that these are systemic issues that extend far beyond this sector [41–43]. The strength of feeling on this subject prompted the current authors to undertake a subsequent study within this overall research project, which will consider career outcomes as at August 2024 for early career researchers who received master’s or PhD funding for canine-relevant health and welfare research between 2012 and 2018. Similarly, there was a strong consensus that the burden of grant writing is a huge temporal challenge for senior researchers, which occupies time that could be better spent elsewhere, and that they would find great value in reform to reduce duplication of effort by standardising application processes across funders and by providing more feedback on failed applications to inform resubmissions. These issues are addressed further by the current authors in a subsequent report, currently in preparation.

Variations on the Delphi research approach are now widely used within health sciences because of their value for investigating the current state of knowledge or opinion in fields that cannot be studied experimentally [7]. Critical appraisals of the Delphi approach note that the methodologies employed vary between studies in their investigative rigor [11]. However, although ‘expert opinion’ is situated on the lowest level of the evidence pyramid, the Delphi approach is nevertheless widely regarded as a valuable tool for investigating the range and direction of informed understanding within a field, particularly with regard to real-world problems [11,44]. Therefore, variations on the Delphi approach are commonly used to investigate complex problems such as determining policy and setting future goals, making it a suitable methodology for the current study [10].

Any research that employs a Delphi approach must balance investigative rigor with practicality. The current study was designed as a modified group Delphi with two rounds of participation. Although iterative discussion is central to the Delphi methodology, the logistical issues of cost, time and participant attrition mean that, in one previous metanalysis, the majority (90%) of Delphi studies surveyed employed two or three rounds of debate, with 48% involving two rounds, like the current study [8]. However, the current study included more participants than most Delphi studies. Between 55 and 60 people contributed to each round of the current study (although not all points of concern in the second round were considered by all participants), whereas 78% of Delphi studies in the previous metanalysis involved 50 or fewer participants [8]. Moreover, in the current study, there was a very low rate of attrition between the two rounds. While this is partly explained by the short duration of the study process, informal conversations with the participants also revealed that many, particularly those working in research, were highly motivated to contribute, both because of their personal enthusiasm for advancing canine health and welfare and because they had strong views about issues in the research sector which they wanted to ensure were included in the analysis. This high motivation was also visible in the 95% response rate observed in the first online participation round.

In this first round, points of concern were suggested freely by participants, then condensed and collated with minimal editorial alteration into structured parts for discussion and prioritisation at the in-person workshop. This approach had advantages and disadvantages. It resulted in a large set of 202 points of concern, which helped to generate rich and comprehensive discussions, where eventual consensus was sometimes facilitated by iterative conversations that returned to similar topics repeatedly. However, with so many overlapping points, few discussion groups fully covered all the material during the available time.

Study participants were deliberately selected to encompass a wide range of expertise in the canine health and welfare sector. Among those who completed the demographic information in the pre-workshop questionnaire, 78% were female, which is likely to be broadly representative of the overall workforce in this sector, given the high proportion of women in the British veterinary profession (64% in 2021) and the even higher proportion in related occupations such as veterinary nursing (97% in 2021) [45]. Despite efforts to recruit more younger participants at the beginning of their careers, only one participant was aged under 25 years. This was partly because the in-person workshop took place in early September, when RVC students were mostly unavailable, and also reflects that the pathway to veterinary and/or doctoral qualification is so extended that even early career researchers are usually aged over 25 years. However, the deliberate efforts to recruit participants across veterinary, research, funder, welfare and breeding sectors, and to balance these perspectives across discussion groups, were largely successful. All academic career stages were represented, ranging from several full professors to an MSc student (and senior researchers also recalled and empathised with the experiences of junior researchers, thus representing their perspectives).

Moreover, partly because many participants offered multiple skillsets, the quality of the discussions were rich and nuanced. Most participants had at least ten years of experience in the canine health and welfare sector, and many commented on changes that they had personally noticed during their careers, either as societal attitudes to canine health and welfare have shifted over time or as genuinely new issues have arisen. For example, in discussions about possible veterinary overtreatment and euthanasia, multiple groups noted that this ‘*has become more a problem over the years … and … that young vets have difficulty in managing client expectations’*; similarly, multiple groups commented on the ‘pandemic puppy’ phenomenon, describing subsequent problems with poorly-socialised young dogs that are rehomed, overwhelming rescue centres—a major new issue at the time of the workshop. Discussion of both these issues led to overall high prioritisation of the related points of concern, showing that participants brought reflective and topical insights to their decision making.

In general, participants across all types of expertise made insightful responses to the questions in the online Delphi round that preceded the workshop, suggesting a wide and comprehensive range of points of concern between them. However, among the 28 respondents who had never worked in research, 14 (50%) answered ‘don’t know’ to one or more question/s about research and its funding. This offered useful insight that even otherwise expert stakeholders within the canine health and welfare sector may lack knowledge about research processes if not personally involved. This was reflected during the workshop; during the discussion sessions, researchers sometimes had to explain issues that other participants were entirely unaware of, such as the reluctance of some universities to accept charitable research funding because of its exemption from overhead costs, for example.

Since all participants in the current study were actively involved in the UK canine health and welfare sector at the time of the Delphi study, the knowledge and experience which informed their contributions were inevitably skewed towards currently UK-relevant topics, such as exotic diseases linked to importation or the overarching problems with the breeding and supply of puppies. Therefore, although some UK-based not-for-profit funders of canine-relevant research do direct considerable resources towards international concerns such as the control of rabies [5], such topics were seldom mentioned in the discussion transcripts. Consequently, the priorities identified in the current study might differ significantly in other regions, particularly in developing countries where the trade in dogs is less lucrative, cultural husbandry practices vary and patterns of enzootic disease are dissimilar from the UK [46–48]. However, in other affluent regions where patterns of dog ownership are similar to the UK, such as Western Europe, North America and Australasia, it is likely that similar concerns might be identified: prior research has already identified comparable human attitudes to extreme conformation in dogs across multiple developed countries, for example [28,49–51].

Because workshop participants were asked to observe Chatham House rules (‘participants are free to use the information received, but neither the identity nor the affiliation of the speaker(s), nor that of any other participant, may be revealed’), they could participate in a personal capacity informed by their own experiences and beliefs, rather than feeling compelled to adhere to their official organisational viewpoints, although many participants also referred to official viewpoints and contributed insights informed by the perspectives of their organisational colleagues, thus enriching group discussions and further validating the prioritisation process [23]. Consequently, discussion transcripts sometimes captured participants finding surprising congruence across expected sector divisions; for example, front line charity workers agreeing with people from the show dog world about breeding practices. The variety of expertise within each group meant that points of concern were often considered by people with high expertise on that topic in dialogue with others who were ‘informed outsiders’ and therefore challenged the established thinking, allowing useful and novel insights to be generated and captured. Nevertheless, the tone of discussions was invariably respectful and accepting, although sometimes impassioned. Some critics of the group Delphi format have argued that power imbalances can cause weaker participants to be manipulated or bullied into consensus [7]. Participants in the current study generally spoke with confidence and engaged freely in robust debate, however. The transcripts recorded multiple participants who consciously engaged collaboratively during the discussion process, as shown in the direct quote used in the title of this paper, ‘*Maybe we should think outside the box*?’ The in-person format allowed participants to provide contextual justification for their viewpoints and to modify individual points of concern which they considered problematic, arguably facilitating an eventual output with more nuance than a questionnaire-only format would have achieved. Moreover, both within the transcripts and in the post-workshop feedback survey, some participants expressed satisfaction at benefiting from the opportunity to speak openly with differently skilled sector experts whom they might not otherwise have met, thus demonstrating the value of open communication between stakeholder groups even during the study process itself.

Metanalyses of Delphi methodologies often challenge how consensus is agreed and evaluated [8,10]. In the current study, a combination of quantitative and qualitative techniques was used to capture both consensus and dissent. For each point of concern, the overall tenor of discussions across all groups was captured and summarised in a short entry on sheets A-D in S1 Dataset, which described what consensus opinion was reached. This format also captured dissent where it occurred, both by noting where recorded verbal feedback was used to modify the original points of concern (for example, changing ‘unregulated online trading’ to ‘lack of regulation or regulatory enforcement for online trading’) and by noting where there was disagreement within or between groups. For example, although participants agreed that ‘humanisation of dogs’ is a common phenomenon, there was no between-group consensus on whether this is a priority issue that impacts canine health and welfare. This lack of consensus was coded both qualitatively in the discussion summary and quantitatively, since this point of concern received all possible priority scores from 1–4 across the seven discussion groups.

Despite the degree of inevitable subjectivity inherent to the Delphi process, the number of participants and the overlap in points of concern meant that the priority scoring process was, in general, fairly rigorous, with sufficient nuance in the transcripts to overcome some problems with the original wording of the points of concern. Time restrictions prevented all discussion groups from considering all points of concern. However, at least two groups (thus a minimum of about 15 people, depending on group size) discussed each point, and 94% of points were considered by at least 3 groups. This, combined with the overlapping content across points of concern, means that most topics were considered in depth by multiple groups.

Because all group priority scores for each point of concern were averaged to create a mean score across all participating groups, mean priority scores of ≤ 2.0 inherently revealed which points of concern had universally been scored higher priority, whereas points of concern with low between-group consensus, or which were rated lower priority by all participating groups, inevitably received larger mean priority scores (> 2.0). Points of concern which did not achieve consensus high priority were consequently excluded from later analysis by the 2.0 priority score cut-off. However, fuller information on these lower-priority points of concern and the accompanying scores are provided in the raw data in the S1 Dataset, and also offer valuable insights. This material ranges from medium priority points that only just missed the 2.0 cut-off for inclusion in the high priority groups (for example, poor husbandry of breeding dogs (score 2.14) or active cruelty such as physical punishment or mutilation (score 2.17)) to low priority points that were genuinely considered unimportant by almost all participants, such as limitations of veterinary technology (score 3.86, generally considered neither a limitation nor lacking) or duplication of research that has already been undertaken (score 4.0, generally agreed that repeatability of research is highly desirable in the medical world and thus likely to be undervalued in the veterinary sector).

In summary, this novel study provided broad insight into current problems in canine health and welfare, indicating highest priority topics for more funding and investigating which topics have been relatively underfunded in the past. Overall, there was a strong emphasis on real-world problems and research with practical impact, with more attention directed towards aspects of the human-canine relationship than on clinical disease. Communication across stakeholder groups, outreach and impact were also prioritised highly, with emphasis on increasing collaboration to maximise the effectiveness of research in advancing canine health and welfare while also supporting the careers and wellbeing of researchers and other human stakeholders involved with canine lives. A subsequent report by the same authors will summarise the overall outputs of the complete research project from which the current study is drawn, and will translate the findings described in the current paper into possible pathways to sector reform, discussing how the highest priorities for change revealed here could be implemented through practical innovations in research practices.

## 5. Conclusions

This Delphi study drew on the expertise of 59 UK-based participants involved professionally and personally with the companion dog sector in various ways to determine which points of concern they deemed of highest importance to canine health and welfare, its research, and to the funding and infrastructure of this field. The themes that emerged from these discussions centred on the importance of human factors, both in driving issues in canine health and welfare and in influencing how research initiatives are structured and funded. Participants recommended that engagement and real-world impact should be integral to the design of research projects, where appropriate. Topics considered highest priority for increased future research funding included the breeding and supply of dogs, topical issues that may need urgent investigation via reactive funding, such as the cost-of-living crisis, specific real-world problems that may not fall into disciplinary specialisms, and common chronic diseases that may be overlooked because of their ubiquity, such as anal sac disease and periodontal disease. There was an overarching emphasis on the benefits of collaboration and transparency in ensuring the optimal deployment of resources throughout the canine research funding sector. Overall, this study provides broad and informed insights about the overall highest priorities for change in UK canine health and welfare research and may therefore usefully inform future reform and innovation in this sector. A subsequent report by the current authors will provide further commentary and suggestions for how the suggested priorities for change in UK canine health and welfare research could be practically achieved.

## Supporting information

S1 Appendix (DOCX)

S1 Dataset (XLSX)

## References

[1] PDSA/YouGov. PAW: PDSA Animal Wellbeing Report 2023. People’s Dispensary for Sick Animals; 2023.

[2] Yeates JW. Maximising canine welfare in veterinary practice and research: A review. The Veterinary Journal. 2012;192(3):272–8. doi: 10.1016/j.tvjl.2011.10.024 22196830

[3] Buckland EL, Corr SA, Abeyesinghe SM, Wathes CM. Prioritisation of companion dog welfare issues using expert consensus. Animal Welfare. 2014;23(1):39–46.

[4] Summers JF, O’Neill DG, Church D, Collins L, Sargan D, Brodbelt DC. Health-related welfare prioritisation of canine disorders using electronic health records in primary care practice in the UK. BMC Veterinary Research. 2019;15(1). doi: 10.1186/s12917-019-1902-0 31118035 PMC6532203

[5] Skipper AM, Packer RMA, O’Neill DG. Researcher, research thyself? Mapping the landscape of canine health and welfare research funding provided by UK not-for-profit organisations from 2012–2022. PLOS ONE. 2024;19(5):e0303498. doi: 10.1371/journal.pone.0303498 38781269 PMC11115267

[6] Linstone HA, Turoff M. The delphi method: Addison-Wesley Reading, MA; 1975.

[7] Niederberger M, Spranger J. Delphi technique in health sciences: a map. Frontiers in public health. 2020;8:561103. doi: 10.3389/fpubh.2020.00457 33072683 PMC7536299

[8] Diamond IR, Grant RC, Feldman BM, Pencharz PB, Ling SC, Moore AM, et al. Defining consensus: A systematic review recommends methodologic criteria for reporting of Delphi studies. Journal of Clinical Epidemiology. 2014;67(4):401–9. doi: 10.1016/j.jclinepi.2013.12.002 24581294

[9] Okoli C, Pawlowski SD. The Delphi method as a research tool: an example, design considerations and applications. Information & Management. 2004;42(1):15–29.

[10] Hsu C-C, Sandford BA. The Delphi technique: making sense of consensus. Practical assessment, research, and evaluation. 2007;12(1).

[11] Shang Z. Use of Delphi in health sciences research: A narrative review. Medicine (Baltimore). 2023;102(7):e32829. doi: 10.1097/MD.0000000000032829 36800594 PMC9936053

[12] Gustafson DH, Shukla RK, Delbecq A, Walster GW. A comparative study of differences in subjective likelihood estimates made by individuals, interacting groups, Delphi groups, and nominal groups. Organizational Behavior and Human Performance. 1973;9(2):280–91.

[13] Panwar R, Hansen E, Kozak R. Evaluating social and environmental issues by integrating the legitimacy gap with expectational gaps: An empirical assessment of the forest industry. Business & Society. 2014;53(6):853–75.

[14] Kim S, Ji Y. Gap analysis. The international encyclopedia of strategic communication. 2018;8:1–6.

[15] Golden SH, Daniel Hager M, Gould LJ, PMP N, Mathioudakis M, Pronovost PJ. A Gap Analysis Needs Assessment Tool to Drive a Care Delivery and Research Agenda for Integration of Care and Sharing of Best Practices Across a Health System. Jt Comm J Qual Patient Saf. 2017;43(1):18–28. doi: 10.1016/j.jcjq.2016.10.004 28334581 PMC6214354

[16] Farooq R. A framework for identifying research gap in social sciences: Evidence from the past. IUP Journal of Management Research. 2017;16(4):66–75.

[17] Thorp TN, Fischer EC, Sinha A. A Delphi study to identify and prioritize research gaps for the incorporation of a fire into life cycle assessment of structures. Fire Safety Journal. 2022;129:103571.

[18] Thompson A, Brennan K, Cox A, Gee J, Harcourt D, Harris A, et al. Evaluation of the current knowledge limitations in breast cancer research: a gap analysis. Breast Cancer Research. 2008;10:1–25. doi: 10.1186/bcr1983 18371194 PMC2397525

[19] Wemelsfelder F. The scientific validity of subjective concepts in models of animal welfare. Applied Animal Behaviour Science. 1997;53(1–2):75–88.

[20] Bennett RM, Broom DM, Henson SJ, Blaney RJP, Harper G. Assessment of the impact of government animal welfare policy on farm animal welfare in the UK. Animal Welfare. 2004;13(1):1–11.

[21] Rioja-Lang F, Bacon H, Connor M, Dwyer CM. Prioritisation of animal welfare issues in the UK using expert consensus. Veterinary Record. 2020;187(12):490-. doi: 10.1136/vr.105964 32631849 PMC7848064

[22] Berteselli GV, Messori S, Arena L, Smith L, Dalla Villa P, de Massis F. Using a Delphi method to estimate the relevance of indicators for the assessment of shelter dog welfare. Animal Welfare. 2022;31(3):341–53.

[23] Chatham House. Chatham House Rules. 2024.

[24] O’Neill DG, James H, Brodbelt DC, Church DB, Pegram C. Prevalence of commonly diagnosed disorders in UK dogs under primary veterinary care: results and applications. BMC Veterinary Research. 2021;17(1):69. doi: 10.1186/s12917-021-02775-3 33593363 PMC7888168

[25] Mattin MJ, Boswood A, Church DB, López-Alvarez J, McGreevy PD, O’Neill DG, et al. Prevalence of and Risk Factors for Degenerative Mitral Valve Disease in Dogs Attending Primary-care Veterinary Practices in England. Journal of Veterinary Internal Medicine. 2015;29(3):847–54. doi: 10.1111/jvim.12591 25857638 PMC4895395

[26] Furtado T, Rogers S, White J. Animal advocacy and human behavioural change. Routledge Handbook of Animal Welfare: Routledge; 2022. p. 467–79.

[27] Reed K, Upjohn MM. Better Lives for Dogs: Incorporating Human Behaviour Change Into a Theory of Change to Improve Canine Welfare Worldwide. Front Vet Sci. 2018;5:93. doi: 10.3389/fvets.2018.00093 29892603 PMC5985712

[28] Bonnett BN, Megens M, O’Neill DG, Hedhammar Å. International and National Approaches to Brachycephalic Breed Health Reforms in Dogs. Health and Welfare of Brachycephalic (Flat-faced) Companion Animals: CRC Press; 2021. p. 127–51.

[29] Guidance: Ban on XL Bully dogs [press release]. 1 April 2024 2024.

[30] CMA identifies multiple concerns in vets market [press release]. 12 March 2024 2024.

[31] Tulloch JSP, Owczarczak-Garstecka SC, Fleming KM, Vivancos R, Westgarth C. English hospital episode data analysis (1998–2018) reveal that the rise in dog bite hospital admissions is driven by adult cases. Scientific Reports. 2021;11(1):1767. doi: 10.1038/s41598-021-81527-7 33469116 PMC7815787

[32] Tulloch JSP, Oxley JA, Christley RM, Westgarth C. Dog-related deaths registered in England and Wales from 2001 to 2021. Public Health. 2023;215:91–3. doi: 10.1016/j.puhe.2022.12.005 36652787

[33] Carri W, Megan B, Robert MC. How many people have been bitten by dogs? A cross-sectional survey of prevalence, incidence and factors associated with dog bites in a UK community. Journal of Epidemiology and Community Health. 2018;72(4):331. doi: 10.1136/jech-2017-209330 29437877 PMC5868524

[34] Christley R, Nelson G, Millman C, Westgarth C. Assessment of Detection of Potential Dog-Bite Risks in the Home Using a Real-Time Hazard Perception Test. Anthrozoös. 2021;34(6):767–86.

[35] SAVSNET. About SAVSNET University of Liverpool2024 [https://www.liverpool.ac.uk/savsnet/about/.

[36] VetCompass. VetCompass: your knowledge hub 2024 [https://www.rvc.ac.uk/vetcompass.

[37] Perry KL, Déjardin LM. Canine medial patellar luxation. Journal of Small Animal Practice. 2021;62(5):315–35. doi: 10.1111/jsap.13311 33600015

[38] O’Neill DG, Hendricks A, Phillips JA, Brodbelt DC, Church DB, Loeffler A. Non‐neoplastic anal sac disorders in UK dogs: Epidemiology and management aspects of a research‐neglected syndrome. Veterinary Record. 2021;189(2):no–no. doi: 10.1002/vetr.203 33645764

[39] O’Neill D, Mitchell C, Humphrey J, Church D, Brodbelt D, Pegram C. Epidemiology of periodontal disease in dogs in the UK primary‐care veterinary setting. Journal of Small Animal Practice. 2021;62(12):1051–61. doi: 10.1111/jsap.13405 34374104 PMC9291557

[40] Niemiec BA. Periodontal Disease. Topics in Companion Animal Medicine. 2008;23(2):72–80. doi: 10.1053/j.tcam.2008.02.003 18482707

[41] Fisher JJ, James JL. Know the game: Insights to help early career researchers successfully navigate academia. Placenta. 2022;125:78–83. doi: 10.1016/j.placenta.2021.10.013 34743918

[42] Brami M, Emra S, Muller A, Preda-Bălănică B, Irvine B, Milić B, et al. A Precarious Future: Reflections from a Survey of Early Career Researchers in Archaeology. European Journal of Archaeology. 2023;26(2):226–50.

[43] Christian K, Johnstone C, Larkins J-a, Wright W, Doran MR. A survey of early-career researchers in Australia. eLife. 2021;10:e60613. doi: 10.7554/eLife.60613 33423739 PMC7800379

[44] Ingham-Broomfield B. A nurses’ guide to the hierarchy of research designs and evidence. Australian journal of advanced nursing: a quarterly publication of the Royal Australian Nursing Federation, The. 2016;33:38–43.

[45] Royal College of Veterinary Surgeons. RCVS Facts: facts and figures from the Royal College of Veterinary Surgeons. Royal College of Veterinary Surgeons; 2021 2021.

[46] Bryce CM. Dogs as Pets and Pests: Global Patterns of Canine Abundance, Activity, and Health. Integrative and Comparative Biology. 2021;61(1):154–65. doi: 10.1093/icb/icab046 33940621

[47] Sykes N, Beirne P, Horowitz A, Jones I, Kalof L, Karlsson E, et al. Humanity’s best friend: a dog-centric approach to addressing global challenges. Animals. 2020;10(3):502. doi: 10.3390/ani10030502 32192138 PMC7142965

[48] Davlin SL, VonVille HM. Canine rabies vaccination and domestic dog population characteristics in the developing world: A systematic review. Vaccine. 2012;30(24):3492–502. doi: 10.1016/j.vaccine.2012.03.069 22480924

[49] Packer RM, O’Neill DG, Fletcher F, Farnworth MJ. Great expectations, inconvenient truths, and the paradoxes of the dog-owner relationship for owners of brachycephalic dogs. PLoS One. 2019;14(7):e0219918. doi: 10.1371/journal.pone.0219918 31323057 PMC6641206

[50] Steinert K, Kuhne F, Kramer M, Hackbarth H. People’s perception of brachycephalic breeds and breed-related welfare problems in Germany. Journal of Veterinary Behavior. 2019;33:96–102.

[51] Sandøe P, Kondrup SV, Bennett PC, Forkman B, Meyer I, Proschowsky HF, et al. Why do people buy dogs with potential welfare problems related to extreme conformation and inherited disease? A representative study of Danish owners of four small dog breeds. PloS one. 2017;12(2):e0172091. doi: 10.1371/journal.pone.0172091 28234931 PMC5325474

