# Supplementary material for: “Maybe we should think outside the box?” prioritisation of issues with UK not-for-profit canine health and welfare research funding using Delphi expert consensus and gap analysis
Source: PLoS One. 2024 Dec 4;19(12):e0313735. doi: 10.1371/journal.pone.0313735 (PMC11616890; doi:10.1371/journal.pone.0313735)
Supplement: S1 Appendix — (DOCX) [file pone.0313735.s001.docx]

**Appendix A**

This appendix contains a more detailed account of the methodology used in this Delphi study, and also includes some of the more detailed results, particularly concerning the demographic characteristics of the study participants. It should be read in conjunction with the main paper.

**Method (detailed)**

1. **Recruitment of study participants**

To develop a collaborative consensus derived from a broad range of stakeholders, an iterative process was used to recruit participants. The intention was to gather approximately 50-60 UK-based people who between them offered broad expertise in canine health and welfare, including veterinary professionals involved in first opinion and referral practice and research; researchers engaged in biological and social sciences and humanities; representatives of funding organisations; people who worked for dog-relevant charitable organisations; and dog breeders. This number of participants was chosen to ensure that all these skillsets would be represented by multiple people (many individual people also offered multiple skillsets, such as veterinary professionals who had worked for several types of organisation). Recruitment began through personal networking by the author team and was then expanded through asking direct contacts (such as senior veterinary staff at charitable organisations) to propose people with other expertise (such as front-line rescue workers) from within their own networks. A tally was kept of recruited participants with different types of expertise; if one invited person was unable to participate, another person with similar expertise was approached in their stead, to ensure a balance of skillsets across the study. Recruitment continued until a few days before the workshop, because participants were added to the list iteratively as they responded to invitations, and because some were approached belatedly to replace people who withdrew at short notice.

1. **Pre-workshop online questionnaire**

Six weeks before the workshop, an anonymous online pre-workshop questionnaire was circulated to all confirmed study participants. The questionnaire was closed one week before the workshop. The questionnaire is given below.

**Pre-workshop questionnaire for attendees**

1. Describe your experience in the canine health and welfare sector, including **any current or previous roles** (check all that apply)
2. Dog owner (current or previous)
3. Dog breeder (have deliberately bred at least one litter)
4. Dog trainer (have received an income from this)
5. Hold a qualification in canine behaviour
6. Employed by a charity or welfare organisation, working with dogs and/or the public
7. Employed by a charity or welfare organisation, working in research
8. Employed by a charity or welfare organisation, working in management
9. Employed by a charity or welfare organisation and involved in funding decisions
10. Providing care for rescue dogs (including both voluntary and professional involvement)
11. Veterinary surgeon
12. Veterinary nurse
13. Clinical staff at a veterinary school
14. Academic or research staff (university) involved in canine related research
15. Academic or research staff (university) not involved in canine related research
16. Employed by a veterinary corporate
17. Employed in veterinary practice (non-corporate)
18. Employed by a non-clinical commercial organisation
19. Employed in a sector unrelated to animals
20. Student
21. Unemployed or retired
22. Involved in competitive canine activities for leisure (dog shows, agility, etc)
23. Other (please specify)
24. Please briefly describe, in your own words, what you consider to be your current key identity(ies) or role(s) in the canine health and welfare sector (for example, ‘kennel worker at rescue centre, veterinary surgeon in general practice, researcher into canine genomics’)

[free text response]

1. What do you think are the biggest problems that currently reduce canine health and welfare?

[free text response]

1. In your opinion, what are the major structural (practical, political, logistical, financial, ethical, etc) problems with the current UK landscape of canine health and welfare research funding (if any)?

[free text response]

1. Are there any areas of canine health and welfare research which you think receive more funding than is necessary?

[free text response]

1. Are there any areas of canine health and welfare research which you think are neglected and receive less funding than is necessary?

[free text response]

1. What changes, if any, would you like to see in the UK funding of canine health and welfare research, in order to increase its positive impact for canine health and welfare?

[free text response]

1. What topics would you like this workshop to discuss, which have not already been mentioned in this questionnaire?

[free text response]

Demographic questions – ‘The following questions are optional’

1. What is your gender?

[male]

[female]

[other]

[prefer to self-describe as]

1. Are you

[18 to 24 years old]

[25 to 34 years old]

[35 to 44 years old]

[45 to 54 years old]

[55 to 64 years old]

[65 to 74 years old]

[75 years old or older]

1. Where do you live?

[Southern England]

[Midlands]

[Northern England]

[Wales]

[Scotland]

[Northern Ireland]

[other]

1. How long have you been active in the canine health and welfare sector?

[free text response]

1. What academic qualifications do you have, if any? Check all that apply

[No post school qualifications]

[Technical qualifications]

[Bachelor’s degree]

[Registerable veterinary qualifications (MRCVS or RVN)]

[Master’s degree]

[PhD]

[other qualifications in animal-related subject]

[other higher qualifications (unrelated to animals)]

1. **Collation of responses from pre-workshop questionnaire**

The demographic information provided by respondents was tabulated in Microsoft Excel. Information about participant skillsets was tabulated separately, using anonymised respondent ID numbers, to separate it from the demographic data. Using Microsoft Word, the free-text responses to the six questions were extracted, deconstructed into individual components, and collated into topics within each question category to create a long list of elicited points of concern. Where two or more respondents had raised points that were very similar or the same, their responses were condensed into one, while still aiming to avoid editorial distortion or loss of nuance through collating responses which were not fully congruent. For example, ‘breeding for extreme conformation/looks’, ‘inbreeding’ and ‘lack of health testing/consideration of inherited disease’ were retained as separate points within the wider umbrella topic of ‘poor breeding practices’, under question category (1): ‘problems that reduce canine health and welfare’.

Following this process, these extracted points of concern were grouped into four parts to structure discussion at the workshop, which corresponded to five of the six questions previously described. These were:

A. What are the most important problems that currently affect canine health and welfare?

B. What are the most important problems with current funding processes?

C. What areas of research are most under- or over-funded?

D. What are the priorities for change?

The sixth question, ‘what topics would you like the workshop to discuss, which have not already been mentioned in this questionnaire?’, was used to supplement responses to the other five questions and to alert the authors to matters of particular interest to certain respondents. For example, one respondent expressed reservations about bias in the Delphi study design, and so this was specifically addressed during the workshop briefing.

These topics and individual points of concern were tabulated in Microsoft Excel within the four discussion parts A-D. The number of respondents who mentioned each topic in their questionnaire answers was counted, and this information was used to rank topics so that more popular topics were prioritised within each discussion part. Within each topic, points of concern were listed alphabetically. This discussion framework (see sheets A-D in S1 dataset) was pre-circulated to all workshop attendees to allow familiarisation with its content. Delegates were informed that the workshop would involve a collaborative ranking process for each point of concern, and were invited to optionally score the lists themselves beforehand to facilitate open and free discussion on the day.

1. **Group workshop**

Immediately before the in-person workshop, expected attendees were sorted non-randomly into six discussion groups. The groups were deliberately selected to ensure as even a spread of expertise as possible between groups: for example, a single group of eight people might include a funder, a clinical researcher, a veterinarian in first opinion practice, a geneticist, a charity worker, a veterinary nurse and a breed health co-ordinator, with individual people potentially representing multiple roles. Attendees from the same organisation were separated across groups where possible. Each group was allocated a session leader, either one of the authors of this paper or one of their postgraduate students or staff colleagues. At the start of the event all attendees were reminded that Chatham House rules applied, so that they could speak freely without their views being attributable to them thereafter.

Attendees were briefed on the purpose and scope of the research. They were then asked to discuss the various parts within their allocated groups and to collaboratively score each point of concern from 1-4, where 1 was highest priority and 4 was lowest priority. Each basis for prioritisation was defined according to the context of that discussion part: for example, in part A the definition was ‘highest priority for negative impact on overall canine health and welfare’, whereas in part B the definition was ‘the most detrimental structural problems in the current UK landscape of canine health and welfare funding’. All groups were asked to discuss parts A and D, in full, because these were considered to cover the topics that were most vital to the key aims of the study. Half the groups were asked to follow-on with a discussion of part B, followed by C if they had sufficient time, while the other half were asked to also discuss C, followed by B if possible. All sessions were recorded in duplicate, via a hand-held voice recording device and the session leader’s phone. Each session leader noted the consensus score for each point of concern on a printed copy of the overall spreadsheet, and additionally noted if there had been particular difficulty in reaching agreement, or any other issue that arose (for example, if the group had challenged the given wording for a particular point of concern).

For various personal reasons, eight delegates were unable to attend the in-person workshop on the day, but nevertheless expressed their willingness to participate in the study. By chance, the spread of expertise across these participants was similar to that within each in-person discussion group, so a supplementary online session was arranged in October 2023 to capture their input, making a total of seven discussion groups available for the final analysis. The online session used the same discussion framework as the in-person workshop, but due to time constraints only addressed parts A and D.

**4.1 Post workshop communication with delegates**

All delegates were invited to complete a post-workshop questionnaire, enabling them to provide feedback on their experience of the event and to raise any ensuing questions.

1. **Post-workshop data processing**

After the workshop, all seven session recordings were manually transcribed by the lead researcher (AMS). Automated transcription using Microsoft Word’s Transcribe function was trialled but proved unsuitable, due to frequently overlapping voices on the recording and the heavy use of specialist vocabulary. Elements from each transcription that concerned each point of concern were extracted, compared across discussion groups, and summarised to a few sentences that captured the overall consensus viewpoint, including any disagreement within or between discussion groups and any instances where attendees challenged the wording of that point of concern. Each point of concern summary and all individual discussion group prioritised scores were entered into one Excel workbook (Microsoft), using one sheet for each discussion part A-D.

A mean priority score was calculated for each point of concern from the scores of all participating discussion groups. In a few cases, this involved post hoc manual editing of scores awarded during the workshop, following prompts in the discussion transcript. For example, where groups were short of time, they sometimes left verbal notes in the recordings asking the researchers to extrapolate scores awarded elsewhere to other similar points of concern. Such edits are noted in the S1 dataset.

Once cross-checking with the transcripts was completed, the discussion groups were randomised and anonymised within each separate spreadsheet to obscure which group produced each score (i.e. the discussion groups were identified by numbers on the day and are labelled with letters in the accompanying dataset, but each number corresponds to a random letter on each sheet; see S1 dataset).

**5.1 Data analysis**

Once all points of concern had been verbally summarised in this way and a mean priority score calculated for each, the overall data were checked and cleaned. The mean priority score data were then scrutinised to determine a suitable cut-off point for higher priority points of concern. Within each discussion part A-D, the range of mean priority scores was assessed for normality by visual inspection of histograms and of normal Q-Q plots using SPSS (IBM). These graphs revealed reasonably normal distributions for all discussion parts except A, which was right skewed with a high frequency of higher priority scores (because 1 = highest priority in this study). Within each discussion part A-D, individual mean priority scores were used to generate overall mean priority scores for each part. These overall mean priority scores were 2.07 (A); 2.41 (B); 2.16 (C); and 2.11 (D). Rather than use a different higher priority cut-off for each discussion part, a pragmatic decision was made to trial a cut-off score of 2.0 across all discussion groups to differentiate between higher and lower priority points of concern.

There were four points of concern which originally received mean priority scores higher than 2.0 (therefore lower priority), where the captured discussions from multiple groups indicated that different wording would have led to higher prioritisation. For example, in discussion part A, point of concern ‘irresponsible or uncommitted ownership’ received a mean priority score of 2.14, but three groups, which all awarded it a priority score of 3.0, indicated that they would have scored it higher priority if the word ‘irresponsible’ had been omitted. In these cases, the wording was revised during this analysis to reflect participants’ concerns, the corresponding individual group scores were altered to a score of 2 to reflect participants’ advised ratings for the revised wording, and the mean priority score was then recalculated accordingly. This amendment produced revised mean priority scores for these four points of <2.0 (higher priority), and so these points were therefore all included as points of higher priority concern.

This process created an overall ‘highest priority’ list of 102 points of concern, 50.5% of the original total number. It was decided that this constituted an appropriate proportion of the original dataset to progress to further analysis, and so the trialled cut-off mean priority score of 2.0 was retained. All points of concern with a mean priority score of > 2 (lower priority) were therefore excluded from further analysis. The remaining highest priority (mean priority score ≤ 2.0) points of concern were re-examined and, where indicated, their wording adjusted to reflect feedback from discussion groups (where this had not previously been done to modify mean priority scores as described above). For example, the points of concern that described regulation of online trading and welfare legislation were altered to mention the importance of regulatory enforcement, in response to participant consensus commentary.

**5.2 Reordering of highest priority points of concern into three sections**

The 102 revised highest priority (mean priority score ≤ 2.0) points of concern were then inductively regrouped into three sections according to the broad focus of each. These were:

1. Research topics in canine health and welfare, which included both dog-facing problems, such as emerging infectious diseases or long-term shelter welfare, and human factors, such as breeding for extreme conformation/looks or the cost-of-living crisis.
2. Research approaches and methodologies, such as ‘prospective studies’ or ‘social science research’.
3. Research processes and infrastructure, such as ‘support for early career researchers’ or ‘visibility of current funding patterns’.

**6. Further processing of the three reordered sections to provide gap analysis of priorities for change**

Each of these three reordered sections was then scrutinised to remove points of concern which were deemed unsuitable for further consideration within that framework, and further processed separately, as described below.

- 1. **Research topics in canine health and welfare**

The highest priority points of concern that concerned research topics in canine health and welfare, including both dog-facing problems and human factors, were scrutinised for overlap, collated if necessary, and divided into inductively determined meaningful problem categories, informed by the discussion transcripts and by logical categorisation of overlapping or related points of concern. In some cases, these points of concern were merged or adjusted in response to comments from participants at the workshop. For example, the original discussion prompt list for canine behavioural problems included anxiety, aggression and poor socialisation as separate points of concern, but there was a strong workshop consensus that these problems were better grouped as one overarching issue, ‘general behavioural problems’, so this was altered accordingly. Where collation was used, the resulting collated points of concern were awarded a collated mean priority score, calculated as the mean of all relevant original mean priority scores.

In order to compare the highest priority research topics in canine health and welfare from the Delphi study with the historical funding dataset created in the first phase of this project, it was also necessary to collate some highest priority Delphi points of concern in order to map them more directly to historical research funding allocation. For example, ‘welfare issues consequent to defensive medicine’, ‘high-profile cutting edge surgical interventions’, ‘long-term medical treatment of animals with declining quality of life’ and ‘reluctance to euthanase’ were all grouped into one overarching collated point of concern, ‘veterinary overtreatment/euthanasia decisions’, for comparison with the historical dataset.

To perform this analysis, the relevant past funding was extracted from the historical dataset and mapped onto each collated highest priority point of concern in the section on research topics in canine health and welfare. Each research grant was only allotted once within each problem category. However, the same research grant could appear more than once if it was deemed relevant to more than one problem category. For example, the problem category of ‘breeding and supply issues’ included the point of concern ‘human preference for extreme conformation’, and therefore all studies concerning this subject were grouped under this point of concern and excluded from other points of concern within this problem category, such as ‘puppy buying behaviours’ or ‘what breeding is ethical’. In contrast, ‘breed related diseases’ and ‘clinical practice’ were separate problem categories, with ‘common chronic diseases’ one point of concern within the ‘clinical practice’ problem category. Therefore, studies concerning mitral valve disease in Cavalier King Charles Spaniels were included within both ‘breed-related disease’ and ‘common chronic disease’, because to have omitted this condition from either of these two problem categories would have underrepresented its overall funding total.

Once past funding had been mapped to all collated points of concern, a priority score ranking and a total past funding ranking were calculated for each collated point of concern. The overall historical funding for each problem category was calculated and split by type of funder, separating wide-scope funders (UK Research and Innovation councils and the Wellcome Trust) from animal-directed funders, to explore and compare what types of highest-priority research topic were supported by each.

SPSS Statistics version 29.0 (IBM) was then used to assess the association between ranked Delphi mean priority scores (collated where appropriate) and ranked past funding expenditure, and thereby identify which highest priority points of concern had previously received less past funding, relative to their priority score, than others.

A different approach was used to extend the analysis for one highest priority point of concern from the Delphi study, ‘common chronic conditions in first opinion practice’. This could be analysed in greater detail because the relevant historical funding data differentiated effectively between different common chronic conditions, such as arthritis and obesity, so that it was possible to compare the historical allocation of funding across multiple common chronic conditions. Therefore, previously published research that calculated the prevalence of different common conditions in first opinion canine practice was used as an additional tool to inform further analysis of this topic (1). That study extracted data from the clinical records of over 22,000 dogs under first opinion veterinary care in 2016. It reported 29 individual level common disorders with an annual prevalence > 1%. The conditions identified in the previous paper were scrutinised to eliminate common acute conditions, such as bite wounds. Signalments with very mixed aetiologies and durations, such as ‘lameness’ and ‘vomiting’, were eliminated because of their heterogenous causes. Some related and frequently chronic signs and conditions, which the previous paper listed separately, were condensed into grouped categories that mapped more effectively onto identified categories in the historical funding dataset, and the individual prevalences of these conditions was added to obtain a group value. Using this method, ten common chronic conditions with a prevalence ≥ 1% were identified. The historical dataset was then interrogated to identify the total funding previously directed to each of these conditions. Further numerical analysis calculated how much of this total funding would have been directed to each condition if it had been distributed as a ‘fair share’ of the total amount in proportion to the previously reported prevalence of that condition. Finally, the percentage of this ‘fair share’ that was actually allocated to each common chronic condition was calculated, thus estimating which common chronic conditions were relatively underfunded in the historical dataset, assuming that funding should be proportional to prevalence.

- 1. **Research approaches and methodologies**
  2. **Research processes and infrastructure**

The methodology used to analyse these sections was fully described in the main paper.

**Results**

Some results from the main paper are discussed in more detail below.

1. **Recruitment of study participants**

Sixty people were selected and invited to complete the online anonymous pre-workshop questionnaire. As described later, almost all these invitees also participated in the in-person or online workshop discussion groups. However, a very few people (≤ 4) may have completed the questionnaire but not participated in a workshop discussion, or vice versa, due to last-minute acceptances and cancellations. Since the questionnaire was anonymous, it was not possible to assess this precisely.

1. **Pre-workshop online questionnaire**

The pre-workshop questionnaire was completed by 57 people, representing a 95% response rate among the 60 people invited. The respondents included 40 women, 11 men, one person who self-declared as non-binary, and five people who declined to provide gender information. The age distribution of the respondents is shown in Figure 1. All age bands from 18-24 to 64-75 were represented, with a peak of 16 (28.1%) respondents in the 35-44 age band. No respondents stated they were over 75, and three declined to answer. Experience in the canine health and welfare sector ranged from under five years to more than 40 years, with a peak of 19 (33.3%) respondents in the 11-20 years band (Figure 2). Although efforts were made to encourage people from other areas to attend, over half the respondents (37 people) lived in Southern England, with the remainder spread across the rest of the UK, excluding Wales (Figure 3): any geographic bias here may reflect the physical location of the workshop (at the Royal Veterinary College in Potters Bar, just north of London) and, to some extent, the physical location of the networks used for recruitment. Respondents were asked about their qualifications: 32 (56.1%) had a bachelor’s degree, 30 (52.6%) had a veterinary qualification (as veterinary surgeons or nurses), 21 (36.8%) had a PhD, and lower numbers had various other qualifications.

**Fig A1 – Age distribution of attendees at workshop**

**Fig A2 – Number of attendees at workshop with different levels of experience in the canine health and welfare sector**

**Fig A3. Geographic distribution of attendees at workshop**

Respondents were also asked about their current and previous involvement in the canine health and welfare sector. These responses are shown in the ‘participant skillset’ sheet in S1 dataset. Considering direct personal involvement with dogs, 48/57 (84%) of respondents had owned at least one dog; 15/57 (26%) had bred at least one litter; 14/57 (25%) had cared for at least one rescue dog, either professionally or as a volunteer; and 9/57 (16%) had participated recreationally in competitive canine activities, such as dog showing or agility competition. Considering professional involvement with dogs, 6/57 (11%) of respondents had earnt an income from dog training and/or had a qualification in canine behaviour. Altogether, 26/57 (46%) of respondents had worked for a charity: 19 (33%) of these people had worked in a frontline role, 11 (19%) had carried out research, 14 (25%) had worked in management, and 12 (21%) had been involved in funding decisions. Just over half (29/57, 51%) of respondents were veterinary professionals. Of these, 26/57 (46%) were veterinary surgeons and 3/57 (5%) were registered veterinary nurses. Among the veterinary surgeons, 7/57 (12%) had worked as clinicians in a veterinary school, 9/57 (16%) had worked in a corporate practice and 15/57 (26%) had worked in an independent practice. Across all respondents, 22/57 (39%) had worked in a university setting in some capacity, and 29/57 (54%) had worked in canine research, at a university and/or in a charity setting. This data confirmed that the respondents had a broad range of relevant expertise in the canine health and welfare sector.

1. **Workshop discussion**

The respondents’ answers to the free text survey questions were processed as described in the main paper to produce 202 individual points of concern, grouped into umbrella discussion topics and divided into four discussion parts A-D, as shown in Table A1 and in the S1 supplementary dataset. This format was used to provide some structure and streamlining to the discussion process. Within each discussion part, topics were ranked according to how many respondents had mentioned a point of concern within that topic, so that discussion began with topics that had attracted more concern from participants (see Table A1). Overall, there were 41 points of concern in discussion part A (current problems with canine health and welfare); 53 points of concern in discussion part B (problems with current funding processes); 64 points of concern in discussion part C (areas of research that are currently over- or under-funded); and 44 points of concern in discussion part D (priorities for future change in research processes).

**Table A1. Numbers of Delphi respondents suggesting individual points of concern (organised into broader umbrella topics) for four discussion parts A-D relating to canine**

**health and welfare research and its funding**

| **A: The most important problems that currently affect canine health and welfare** | | **B: The most important problems with current funding processes** | | **C: Areas of research that are most underfunded or overfunded** | | **D: Priorities for future change** | |
| --- | --- | --- | --- | --- | --- | --- | --- |
| Topic | Number of mentions | Topic | Number of mentions | Topic | Number of mentions | Topic | Number of mentions |
| Ownership issues | 35 | Infrastructure issues | 37 | Underfunded research areas | | Impact | 23 |
| Breeding practices | 34 | Overarching issues | 26 | Clinical | 18 | Collaboration | 23 |
| Societal issues | 34 | Funding misdirected | 21 | Investigative approach | 17 | Research type | 20 |
| Canine physical disease - breed-related | 28 | Funding body issues | 18 | Human Behavioural Change | 16 | Funder processes | 19 |
| Infrastructure issues | 24 | Researcher issues | 12 | Non-clinical | 13 | Applicant needs | 9 |
| Veterinary issues | 14 | Project issues | 10 | Canine behaviour | 12 |  |  |
| Canine behavioural issues | 13 |  |  | Societal | 8 |  |  |
| Legal issues | 9 |  |  | Overfunded research areas | |  |  |
| Canine physical disease -general | 7 |  |  | Particular research topics | 22 |  |  |
| Human behavioural problems | 6 |  |  | Particular research types | 9 |  |  |

Further results are reported in full within the main paper.

**References**

1. O’Neill DG, James H, Brodbelt DC, Church DB, Pegram C. Prevalence of commonly diagnosed disorders in UK dogs under primary veterinary care: results and applications. BMC Veterinary Research. 2021;17(1):69.
